# From Processing to Performance: A Multi-Scale Machine Learning Framework for SBS-Modified Asphalt Gels with Multi-Target Correlation and Explainable AI

**DOI:** 10.3390/gels12090795

**Published:** 2026-09-01

**Authors:** Zongyuan Wu, Xiaoyu Ma, Mengxin Qiu, Chenze Fang, Weizhan Liu, Decai Wang

**Affiliations:** School of Civil Engineering and Communication, North China University of Water Resources and Electric Power, Zhengzhou 450045, China

**Keywords:** SBS-modified asphalt, polymer gel, machine learning, materials gene, multi-target correlation

## Abstract

Styrene–butadiene–styrene (SBS)-modified asphalt is a physical polymer gel system in which SBS forms a three-dimensional elastic network within the asphalt matrix. This network structure governs the rheological and mechanical properties of the material, yet the quantitative relationships among processing parameters, material composition, microstructure, and macroscopic performance remain insufficiently understood. This study proposes a multi-scale machine learning framework to establish processing–composition–structure–performance mappings for SBS-modified asphalt gels. A dataset of 1072 experimental samples was compiled from a gene database and supplementary laboratory tests. Ten input features were used to predict four performance indicators: penetration, softening point, ductility, and viscosity at 135 degrees Celsius. Four machine learning models were developed and compared. The support vector machine with radial basis function kernel achieved the highest accuracy for penetration with an R^2^ value of 0.9997 and for ductility with an R^2^ value of 0.9996. The artificial neural network performed best for softening point with an R^2^ of 0.9996, and extreme gradient boosting for viscosity with an R^2^ of 0.9993. Optuna-based optimization improved the average R^2^ by 2.1% over default configurations. SHAP analysis identified shear temperature, SBS dosage, and SBS particle size as the most influential factors. The framework enables accurate and interpretable prediction of gel properties and provides a data-driven foundation for material design.

## 1. Introduction

Styrene–butadiene–styrene (SBS) block copolymer is one of the most widely used polymer modifiers for asphalt pavement materials [1]. During high-temperature mixing, SBS particles absorb light aromatic and saturate fractions from asphalt and swell in volume [2]. Upon cooling, polystyrene end-blocks undergo microphase separation and aggregate into glassy nanodomains as physical crosslinking nodes, while polybutadiene mid-blocks form flexible bridging chains between these domains [3,4]. This microphase-separated structure constructs a continuous three-dimensional network throughout the asphalt matrix and endows SBS-modified asphalt with the characteristic viscoelastic behavior of thermoplastic elastomer gels [5]. At normal service temperatures, this network provides elastic recovery to resist permanent deformation under repeated traffic loads. At elevated construction temperatures, physical crosslinks temporarily dissociate, enabling favorable thermoplastic fluidity to satisfy paving requirements [6]. Therefore, the fatigue damage evolution and macroscopic performance of SBS-modified asphalt mixtures are closely associated with the stability of this physical gel network [7].

The macroscopic performance of this complex gel system is governed by the coupled interaction of processing parameters, material composition, and microstructural characteristics [8]. Processing parameters directly regulate SBS dispersion and swelling behavior in the asphalt matrix. Shear temperature controls asphalt viscosity and thermal motion intensity of SBS molecular chains, thereby affecting swelling kinetics and diffusion behavior of polymer particles [9]. Shear rate determines mechanical energy input for breaking SBS aggregates and influences particle size distribution and dispersion uniformity of polymer domains [10]. Shear time affects the sufficiency of SBS swelling and the spatial homogeneity of the final gel network structure [11]. Material composition also plays fundamental roles in defining gel network architecture. The chemical fraction composition of base asphalt determines thermodynamic compatibility between polymer modifiers and asphalt media [12]. The molecular architecture of SBS, including linear and star-shaped structures, determines crosslinking density and mechanical properties of the constructed gel network [13]. SBS dosage directly affects polymer phase volume fraction and significantly influences microstructural morphology and high-temperature rheological properties of modified asphalt [14]. Microstructural characteristics further describe the morphology and structural integrity of the gel network. The average particle size of dispersed SBS domains reflects modifier dispersion quality and network continuity [15]. Fourier transform infrared spectroscopic indices, including the polystyrene index ($I_{PS}$), polybutadiene index ($I_{PB}$), and butadiene index ($I_{B}$), provide quantitative characterization of polymer–asphalt interactions and structural integrity of polymer networks [16,17]. However, the quantitative mapping relationships among these multi-scale influencing factors and corresponding macroscopic performance indicators remain insufficiently understood. The strong nonlinearity and multi-factor coupling characteristics inherent in gel formation make traditional single-factor experimental methods and empirical regression models insufficient for systematically describing structure–performance relationships of SBS-modified asphalt systems [18].

Inspired by the Materials Genome Initiative proposed in the early 2010s, modern materials science has gradually shifted from traditional trial-and-error experimentation toward data-driven material discovery and design [19]. This concept integrates high-throughput characterization, structured database construction, and computational modeling to accelerate the development of advanced materials [20]. In asphalt materials research, this strategy has been gradually implemented through the integration of multi-scale experimental data, computational modeling, and database construction [21]. The asphalt materials gene database developed at Southwest Jiaotong University integrates multi-dimensional information covering binder chemical composition, microstructural morphology, rheological properties, and pavement performance [22]. Recent studies have reviewed asphalt material gene databases and emphasized the integration of multi-scale characterization data and computational modeling for performance prediction and intelligent material design [23]. The concept of asphalt genes and their quantitative relationship with rheological properties has also been systematically established, providing a foundation for standardized gene encoding and large-scale material resource construction [24]. These databases provide important support for constitutive relationship analysis, performance prediction modeling, and intelligent formulation design, reducing dependence on experience-driven trial-and-error methods in asphalt material development [25].

Parallel to the development of material databases, machine learning has emerged as a powerful data-driven approach for modeling complex high-dimensional nonlinear relationships in material systems [26,27]. Various machine learning algorithms have been successfully applied to asphalt property prediction tasks in recent years [28]. Hoang et al. employed artificial neural networks to predict penetration, softening point, ductility, and viscosity of graphene oxide-modified asphalt binders, achieving satisfactory predictive accuracy and identifying aging conditions and initial base asphalt properties as key influencing factors [29]. Wu et al. developed four classical machine learning models based on 436 experimental samples to predict high-temperature stability and low-temperature cracking resistance of cold recycled asphalt mixtures, with genetic algorithms adopted for systematic hyperparameter optimization [30]. In rheological property prediction, researchers have developed specialized machine learning models for modified asphalt binders containing polymers, waxes, and other additives [31]. Sadat Hosseini et al. applied optimized ensemble learning approaches to predict asphalt viscoelastic properties and reported prediction accuracy with R^2^ values exceeding 0.90 for certain indicators [32]. Comparative studies have further investigated the performance of single machine learning models and ensemble learning methods for predicting complex shear modulus and phase angle parameters [33]. More recently, Zhang et al. applied deep learning techniques to characterize SBS-modified asphalt microstructures, demonstrating the potential of advanced neural networks for identifying structural features [34]. Artificial neural network models have also been developed to predict frequency- and temperature-dependent dynamic modulus and phase angle, further confirming the effectiveness of machine learning in asphalt rheological property prediction [35,36].

Although machine learning provides powerful prediction capability, model interpretability has attracted increasing attention in materials science and asphalt engineering research [37]. Explainable artificial intelligence techniques, especially the SHAP method based on cooperative game theory, have been widely applied to quantify feature contributions and reveal mechanisms behind data-driven predictions [38]. Researchers have employed SHAP analysis to identify key factors controlling asphalt rheological properties and extract physical information from complex high-dimensional datasets [39]. The combination of SHAP and partial dependence plots has been applied to identify critical influencing features and visualize nonlinear effects in asphalt fracture resistance and dynamic modulus prediction [40]. Ma et al. used SHAP to examine the physical consistency of data-driven predictions for asphalt mixture dynamic modulus, demonstrating that interpretable machine learning can improve model transparency and support mechanistic understanding [41]. Erten and Gürfidan applied SHAP in regression-based performance prediction for asphalt mixture design and identified parameters such as softening point, penetration, and void ratios as important features [42]. These studies demonstrate that explainable machine learning can transform conventional black-box prediction models into transparent tools for guiding rational material formulation design and process optimization [43].

Despite these advances, three critical research gaps remain in SBS-modified asphalt gel materials. First, existing machine learning studies mainly focus on single-scale property prediction, such as binder rheological properties or pavement performance, while few studies integrate processing parameters, material composition features, and microstructural characteristics into a unified multi-scale framework for predicting macroscopic performance of SBS-modified asphalt gels. Second, most existing studies treat different target performance indicators as independent prediction tasks and overlook the inherent physical consistency among multiple properties governed by the same gel network structure. Third, although prediction accuracy has been extensively investigated, the physical interpretability of machine learning models has received comparatively less attention for polymer-modified asphalt systems with complex structural evolution mechanisms.

This study proposes a multi-scale machine learning framework that integrates processing parameters, material composition features, and microstructural genes as input variables to predict four key macroscopic properties of SBS-modified asphalt gels, including penetration, softening point, ductility, and viscosity at 135 °C. The framework conducts multi-target correlation analysis to reveal the intrinsic relationships among the four properties, develops and compares four representative machine learning models with systematic hyperparameter optimization, and employs SHAP analysis and partial dependence plots for comprehensive model interpretation. The objectives are to establish quantitative processing–composition–structure–performance relationships, reveal the common physical basis shared by different properties, and identify key controlling factors governing SBS gel network formation and macroscopic performance. To our knowledge, this study is the first attempt to combine multi-scale material genes, machine learning prediction, and explainable analysis within a unified framework for SBS-modified asphalt gel systems.

The main innovations of this study are threefold. The first innovation is the systematic integration of materials gene database, multi-target correlation analysis and explainable machine learning for establishing multi-scale structure–property relationships of SBS-modified asphalt gels. The second innovation is the multi-target performance correlation analysis that reveals the physical consistency among four macroscopic properties and their common dependence on gel network integrity. The third innovation is the comprehensive comparison of hyperparameter optimization methods, including Optuna [44], genetic algorithm and default configurations, which provides methodological guidance for selecting appropriate optimization schemes in asphalt materials machine learning studies. To the best of our knowledge, this is the first study to combine these elements in a unified framework for SBS-modified asphalt gels. The proposed framework not only enables accurate performance prediction but also provides interpretable scientific insights that can guide the rational design of high-performance SBS-modified asphalt gel materials.

## 2. Results and Discussion

### 2.1. Results of Multi-Target Performance Correlation Analysis

**Figure 1:**
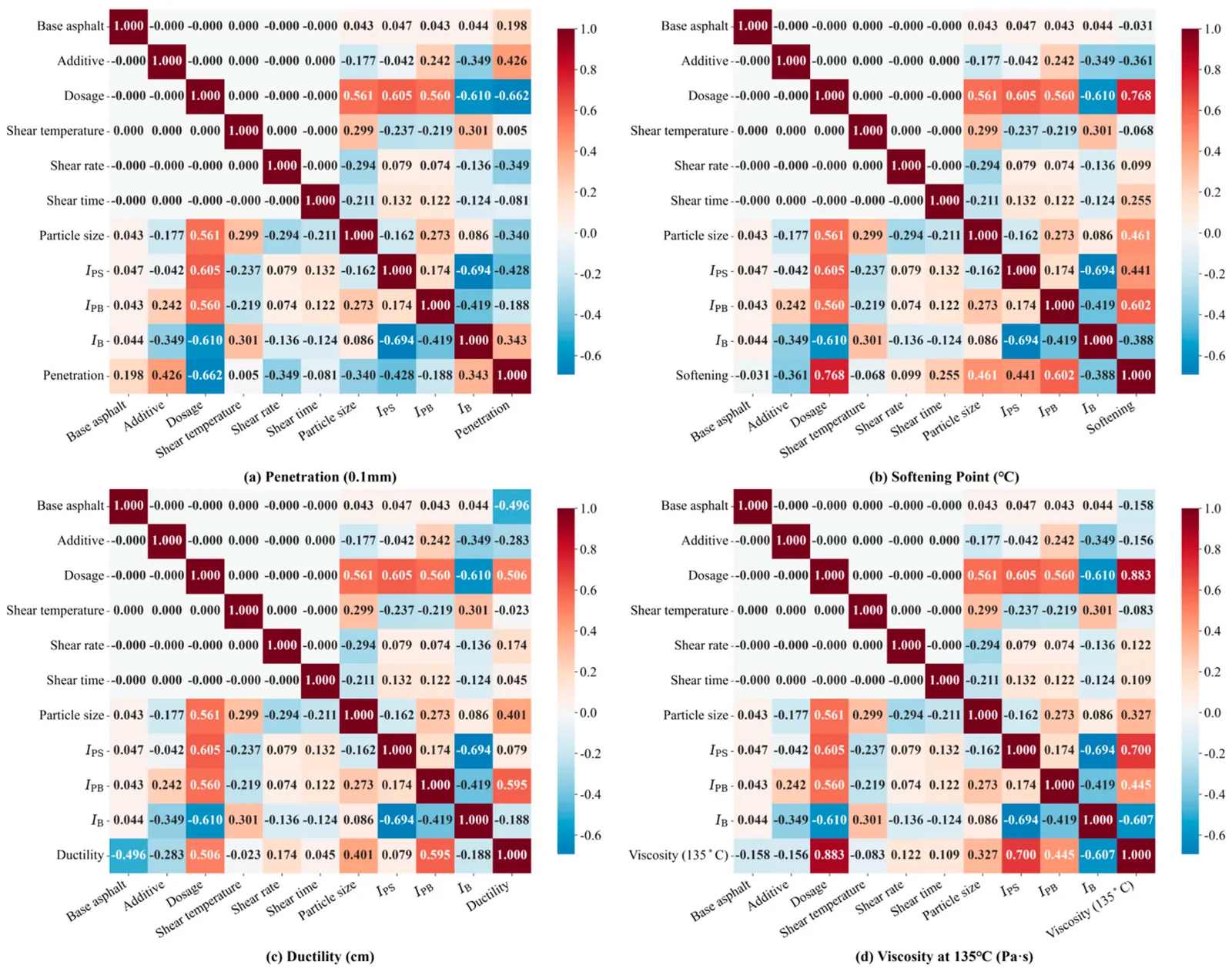
Pearson correlation heatmap of all input features and four target properties. Colors range from deep blue through white to deep red. Color intensity and circle size both indicate correlation strength.

Prior to machine learning modeling, systematic correlation analysis was performed to reveal the inherent physical relationships among target properties and validate the physical consistency of the dataset. Figure 1 presents the Pearson correlation heatmap of all input features and target variables, providing an overview of linear association strength across all variables.

**Figure 2:**
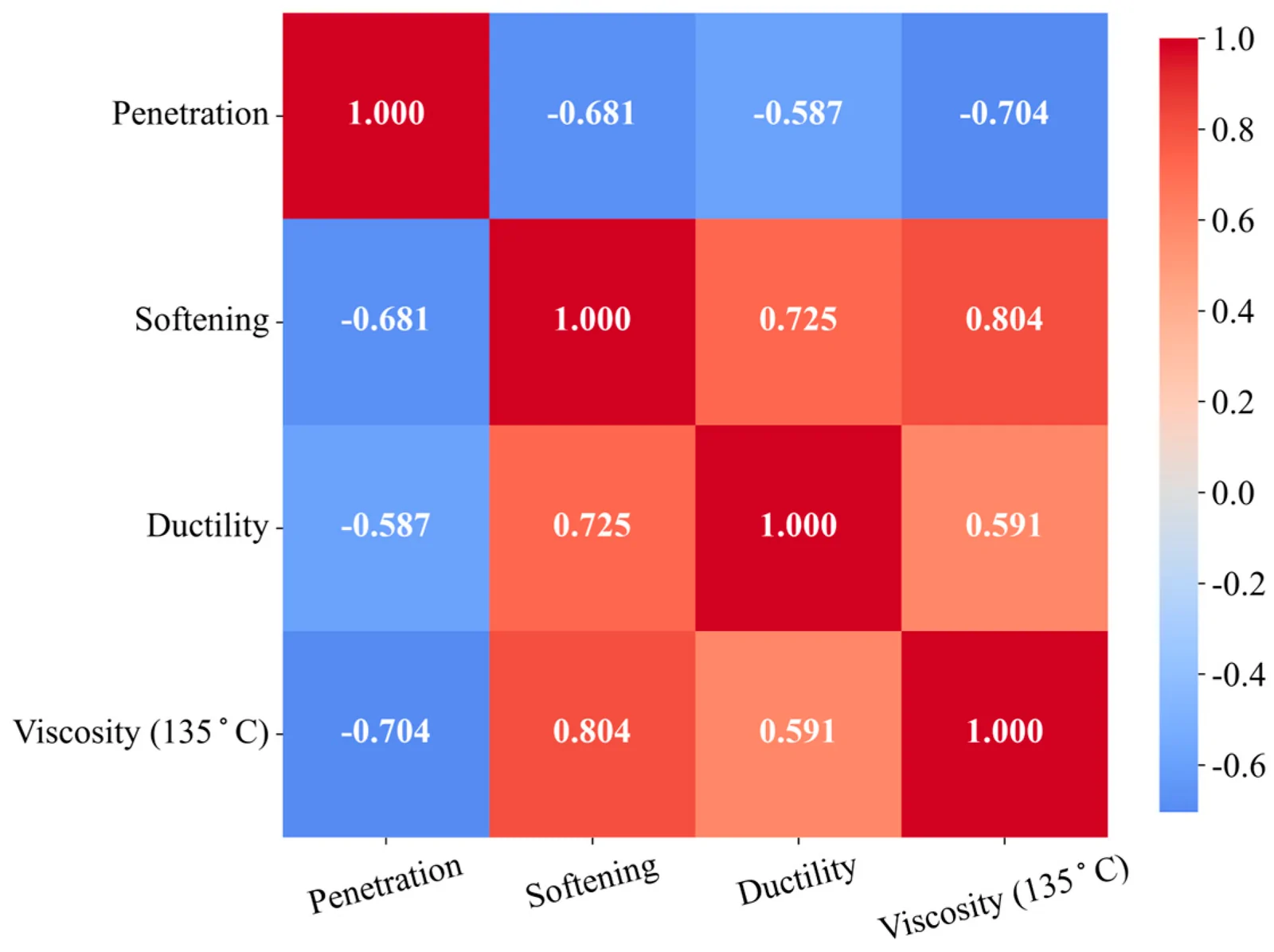
Correlation matrix of the four target properties. Numerical values are Pearson correlation coefficients. All correlations are statistically significant at *p* < 0.001.

Figure 2 shows the correlation matrix focusing exclusively on the four macroscopic target properties. Quantitative results show that penetration has a strong negative correlation with softening point, with a correlation coefficient of −0.681. Penetration also shows negative correlations with ductility and viscosity, with coefficients of −0.587 and −0.704, respectively. Softening point exhibits strong positive correlation with ductility and viscosity, with coefficients of 0.725 and 0.804, respectively. Ductility and viscosity have a moderate positive correlation of 0.591.

These consistent correlation patterns originate from the unified physical mechanism of SBS physical gel systems. All four performance indicators reflect different aspects of the same three-dimensional polymer network structure. A more continuous and robust gel network enhances high-temperature deformation resistance, which manifests as a higher softening point and higher rotational viscosity. A denser network also increases overall system stiffness, leading to lower penetration values. Flexible polybutadiene segments within the network provide elongation capacity under low-temperature conditions, which explains the positive correlation between network integrity and ductility.

Principal component analysis was further applied to the four target variables. The first principal component explains more than 72% of the total variance of all performance indicators. This latent variable can be interpreted as a gel network integrity factor, which quantifies the overall development level of the SBS three-dimensional network. This finding confirms that the four macroscopic properties share a common physical root, and provides a theoretical basis for multi-target collaborative design of SBS asphalt gels.

### 2.2. Model Performance Comparison

Four machine learning models with distinct architectural principles were trained and evaluated through five-fold cross-validation. Predictive performance was quantified from three perspectives: fitting goodness, absolute error, and error dispersion. Table 1 summarizes the mean values of coefficient of determination R^2^, root mean square error RMSE, and mean absolute error MAE for all model-target combinations.

**Figure 3:**
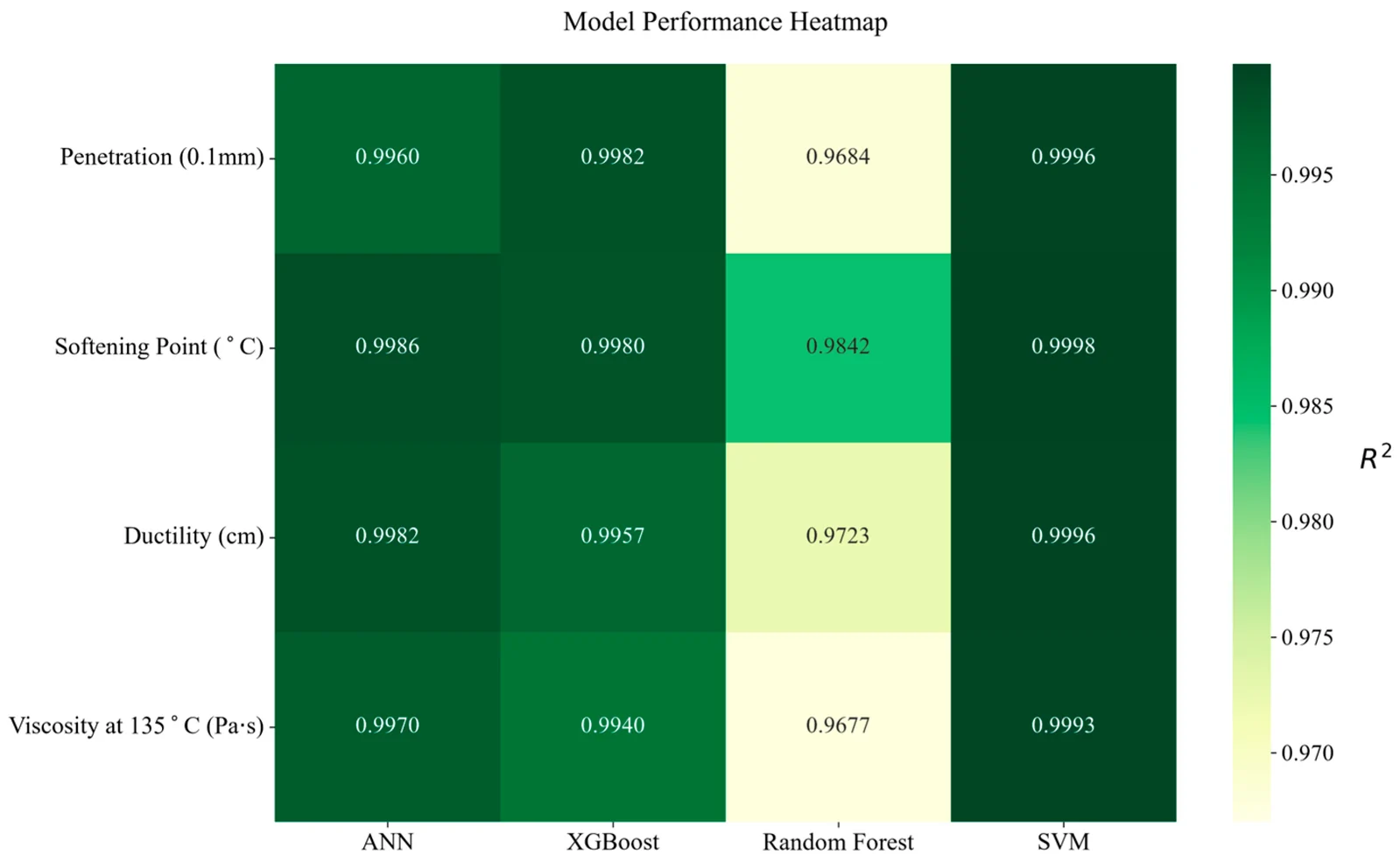
R^2^ performance heatmap of four machine learning models across all target properties.

Figure 3 provides an intuitive heatmap visualization of R^2^ values across all models and targets. Overall, all four models achieve satisfactory predictive accuracy, with R^2^ values above 0.96 for all targets. Most prediction tasks yield R^2^ values above 0.99, indicating that the selected multi-scale input features can fully characterize the determinants of gel macroscopic performance.

Support vector machine achieves the highest prediction accuracy for penetration and ductility, with R^2^ values of 0.9997 and 0.9996, respectively. Artificial neural network performs best on softening point prediction, with an R^2^ of 0.9996. Extreme gradient boosting delivers the most accurate prediction for viscosity at 135 °C, with an R^2^ of 0.9993. Random forest shows slightly lower overall accuracy but still maintains reliable predictive capability for all four indicators.

This performance pattern differs from some previous studies that reported superior performance of ensemble learning methods in asphalt property prediction. The outstanding performance of support vector machine in this work can be explained by two factors. First, the dataset size falls into the medium range, where kernel methods can fully exert their nonlinear fitting capability without suffering from the overfitting risk common in large dataset scenarios. Second, the structure–property relationship of polymer gel systems exhibits relatively smooth nonlinear characteristics. The radial basis function kernel can naturally fit this type of continuous mapping relationship, while tree-based ensemble models are more prone to overfitting local noise in such smooth systems.

**Figure 4:**
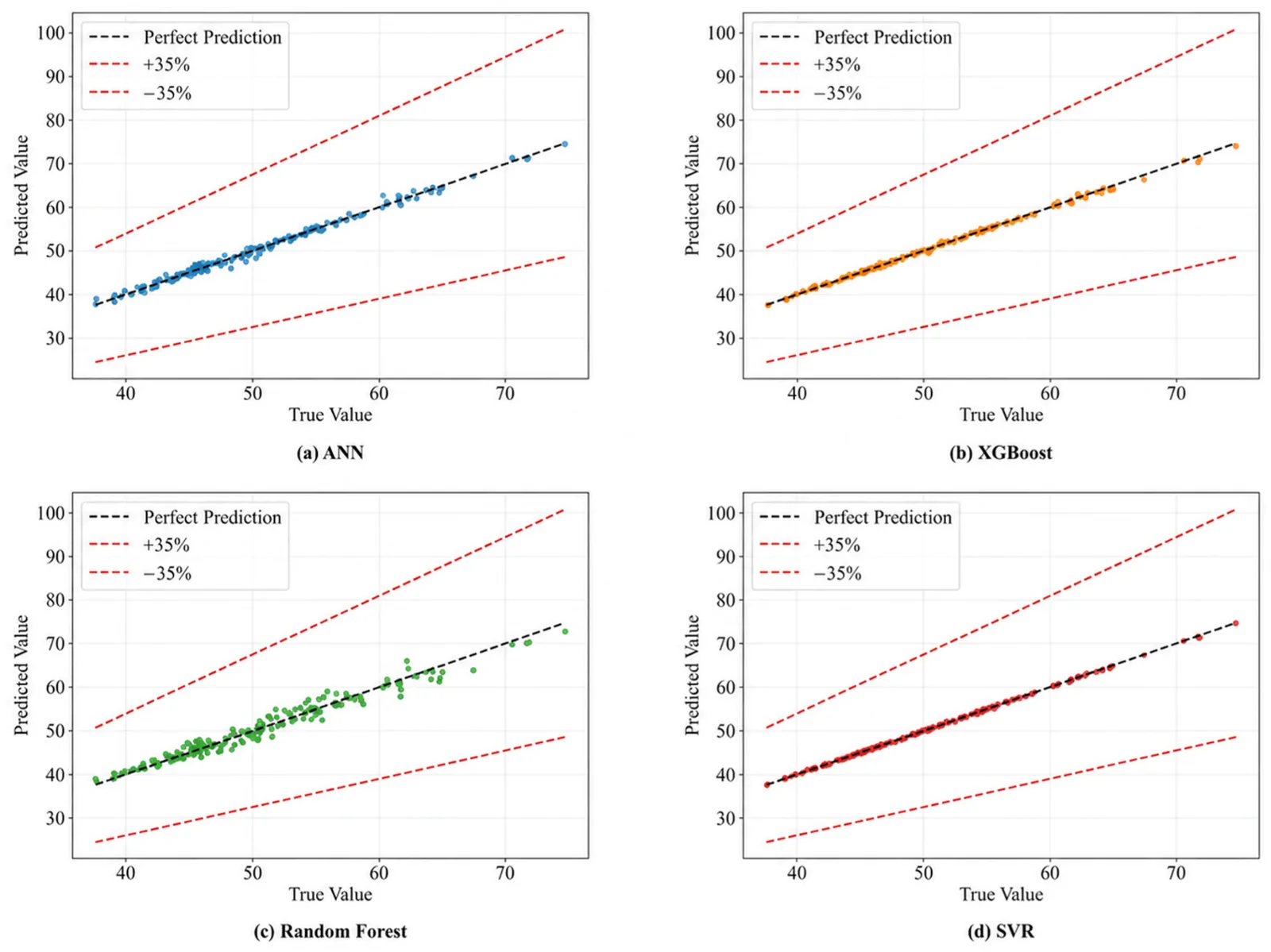
Measured versus predicted values of penetration for the four machine learning models.

**Figure 5:**
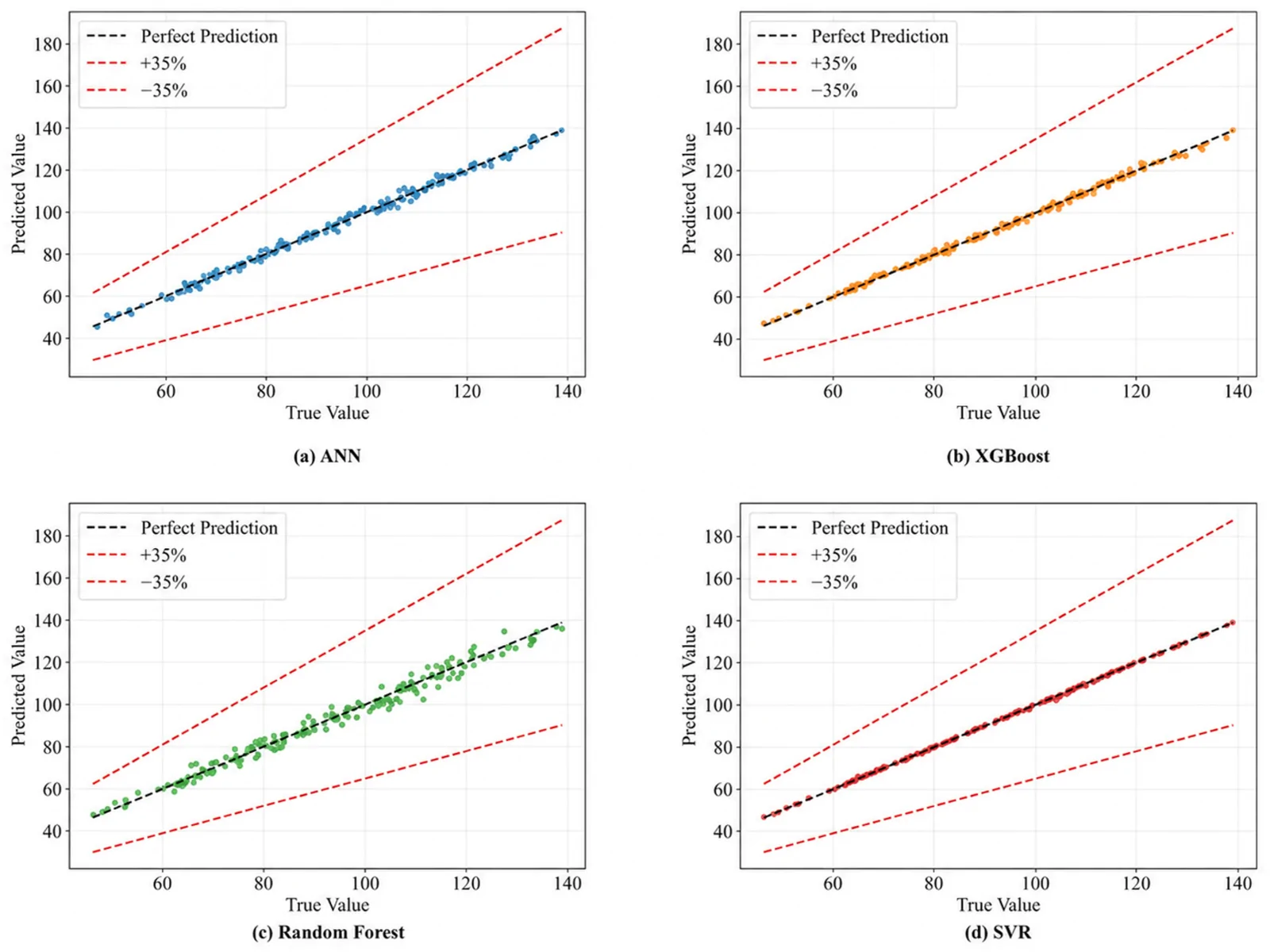
Measured versus predicted values of softening point for the four machine learning models.

**Figure 6:**
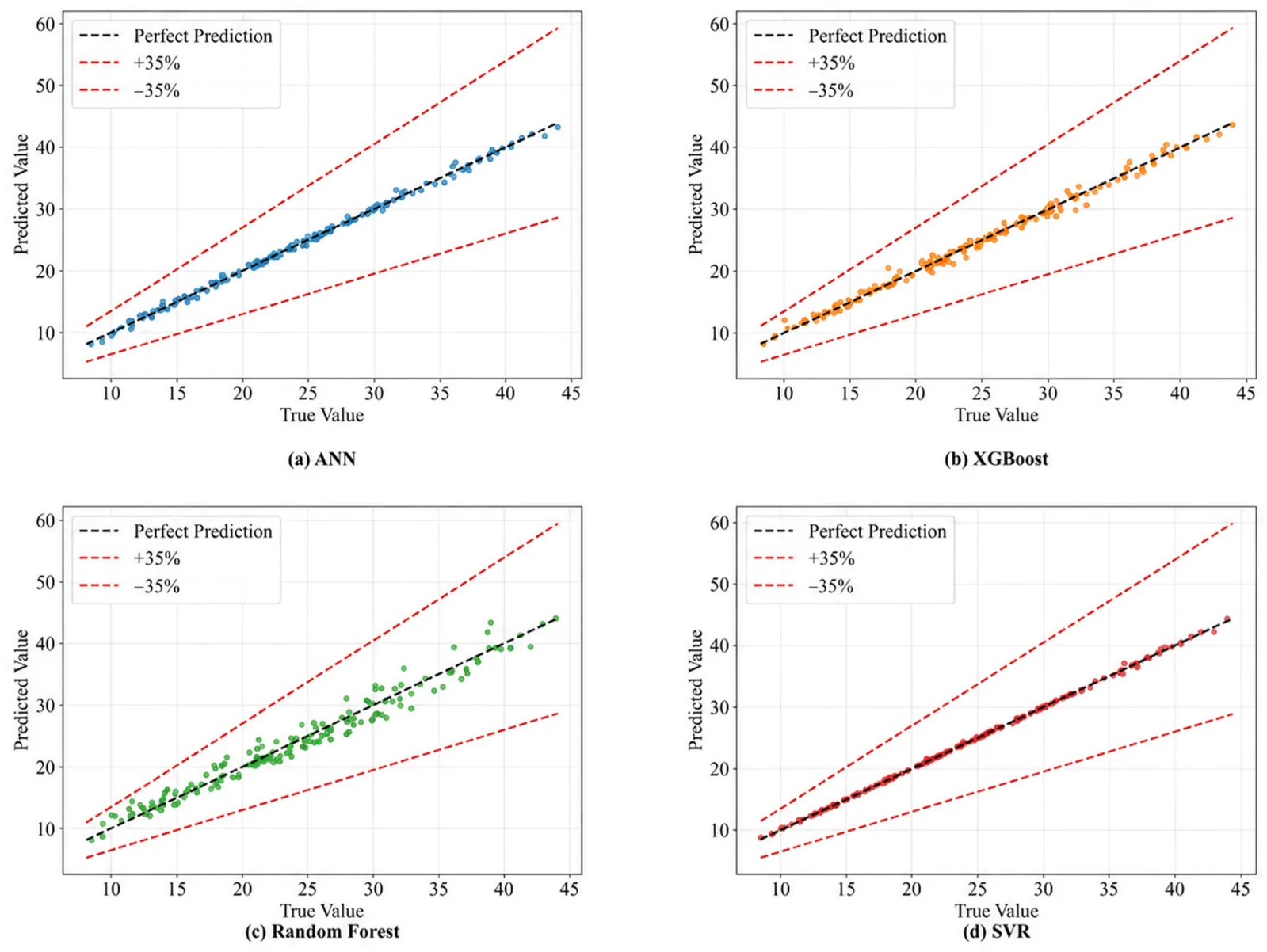
Measured versus predicted values of ductility for the four machine learning models.

**Figure 7:**
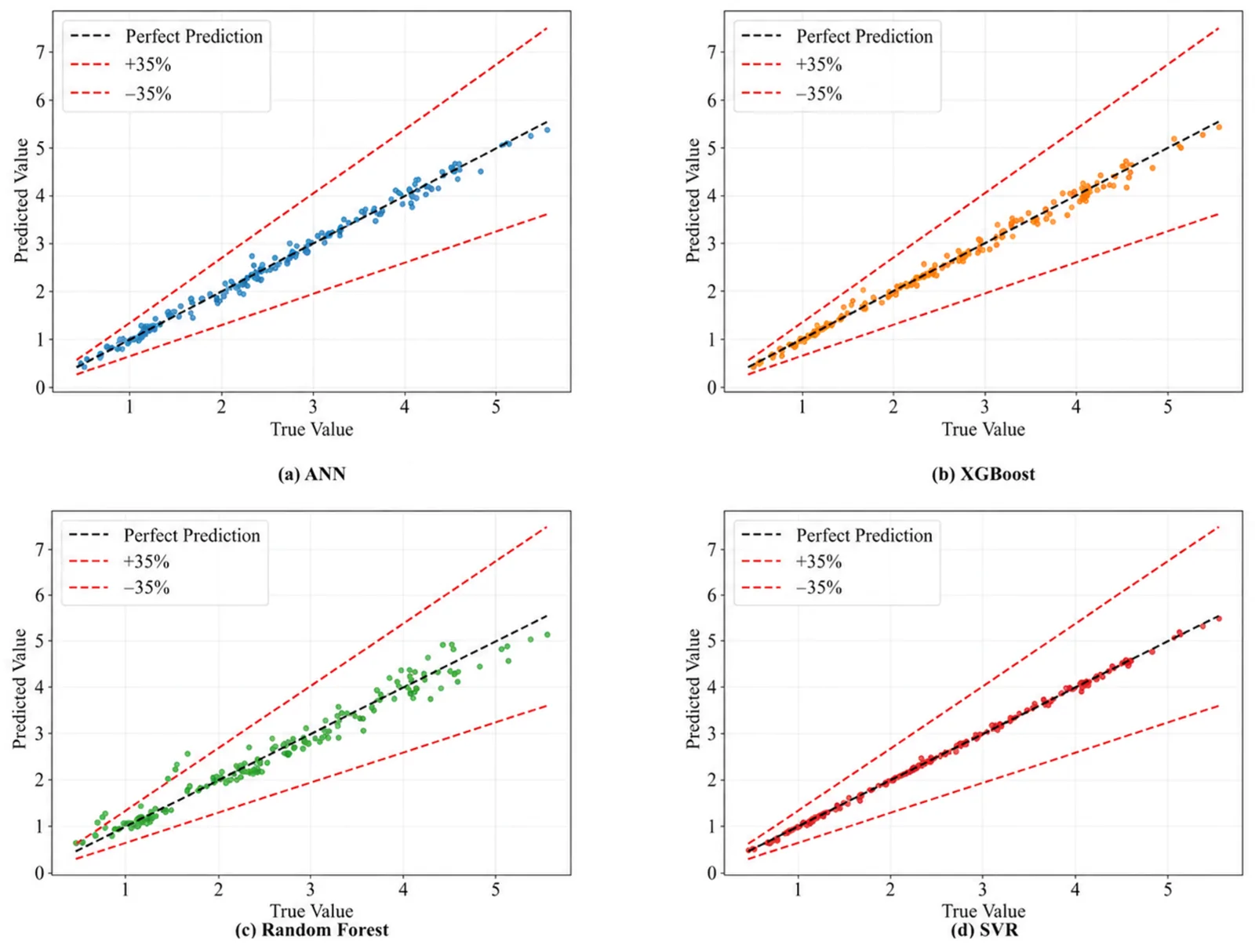
Measured versus predicted values of viscosity at 135 °C for the four machine learning models.

The prediction accuracy achieved in this study compares favorably with previously reported results in the literature. Hoang et al. [29] used artificial neural networks to predict penetration, softening point, ductility, and viscosity of graphene oxide-modified asphalt binders and obtained R^2^ values in the range of 0.92 to 0.96, lower than the 0.9997 achieved by our SVM model for penetration. Wu et al. [30] reported optimal R^2^ values around 0.95 for predicting the high-temperature stability and low-temperature cracking resistance of cold recycled asphalt mixtures using genetic algorithm-optimized models. Sadat Hosseini et al. [32] applied ensemble learning approaches to predict viscoelastic properties of modified asphalt binders and achieved maximum R^2^ values around 0.90. The improved performance in the present study can be attributed to three factors: the systematic integration of multi-scale data from the materials gene database, the joint use of processing, compositional, and microstructural features, which collectively encode more complete structural information, and the intelligent hyperparameter search enabled by the Optuna framework. Direct numerical comparisons with the literature should be interpreted with caution, as input feature sets, data sizes, sample distributions, and testing protocols vary across studies. Nonetheless, the accuracy advantage observed here underscores the effectiveness of multi-scale feature fusion and systematic hyperparameter optimization. Figure 4, Figure 5, Figure 6 and Figure 7 present the measured versus predicted scatter plots for each target property, respectively. All sample points are distributed closely along the diagonal line for all four models, with no obvious systematic deviation across the entire value range. This uniform distribution confirms that all models maintain stable prediction performance for both low and high value intervals, and do not fail at extreme conditions.

Among the four targets, softening point prediction achieves the highest overall accuracy, while penetration and viscosity show slightly larger prediction errors. This difference reflects the inherent stability of different property indicators. Softening point directly corresponds to the glass transition of polystyrene domains in the gel network, and it has a stable one-to-one correspondence with network integrity. Penetration and viscosity are more sensitive to subtle changes in microstructure and test conditions, leading to larger inherent data noise and relatively lower prediction accuracy.

This observation raises an important distinction between two types of prediction uncertainty. The first type stems from model limitations, such as insufficient training data or inappropriate algorithm selection. The second type originates from the inherent variability of the experimental measurements themselves. Penetration testing is known to be highly operator-dependent, with small variations in needle alignment, timing, or sample surface preparation leading to noticeable differences in recorded values. Rotational viscosity measurements are similarly sensitive to spindle selection, temperature equilibration, and sample loading procedures. In contrast, the ring-and-ball method for softening point is more robust and yields highly reproducible results across different operators and laboratories. Therefore, the lower R^2^ values observed for penetration and viscosity do not necessarily indicate model failure. They also reflect the upper bound of predictability imposed by the test methods themselves. This insight has practical implications for future data collection efforts: improving model accuracy beyond the current level would require not only algorithmic advances but also more standardized and precise experimental protocols, particularly for penetration and viscosity tests.

### 2.3. Hyperparameter Optimization Method Comparison

**Figure 8:**
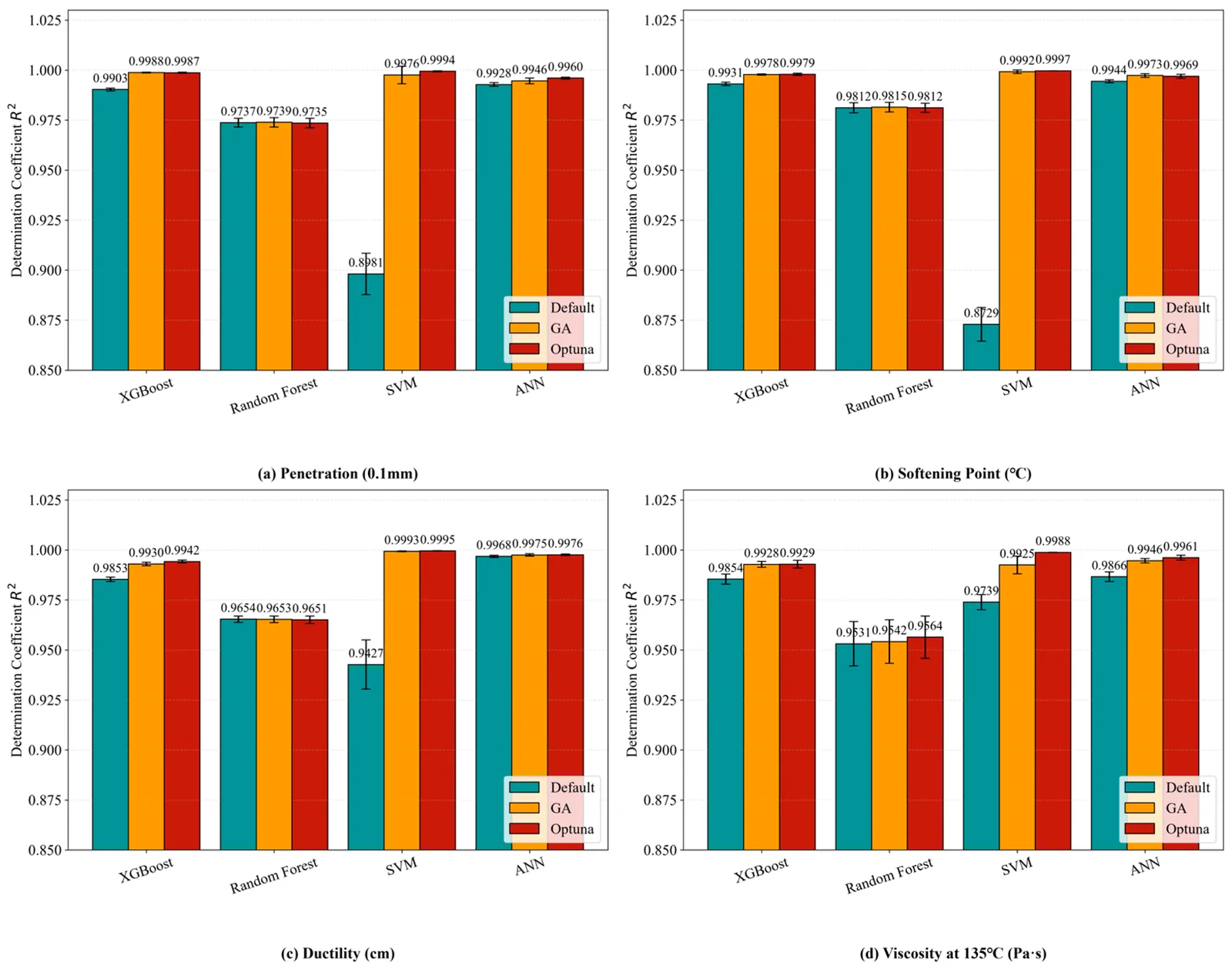
Performance comparison of three hyperparameter tuning strategies across four machine learning models.

Three hyperparameter tuning strategies were systematically compared to evaluate their effectiveness and efficiency for asphalt gel machine learning tasks. Figure 8 shows the R^2^ comparison of default configuration, genetic algorithm optimization, and Optuna optimization across all four models and four targets.

Quantitative analysis shows that both optimization methods improve model performance compared with default parameter settings. Optuna optimization delivers an average R^2^ improvement of 2.1% across all cases, while genetic algorithm optimization achieves an average improvement of 1.7%. The improvement magnitude varies across different model types. Support vector machine and artificial neural network are more sensitive to hyperparameter settings and gain more obvious performance improvement after optimization. The maximum improvement for these two models exceeds 10%. Random forest and extreme gradient boosting have stronger inherent robustness, and their default configurations already deliver decent performance. Optimization brings relatively smaller gains for these two ensemble models.

Table 2 lists the computational cost of each tuning strategy measured by total running time. The default parameter configuration has the lowest computational cost, with training time below 30 s for all models. Genetic algorithm requires multiple generations of iterative evaluation with cross-validation, leading to the longest computational time. All models take more than 600 s to complete genetic algorithm optimization, and random forest optimization takes up to 1116 s. Optuna adopts a tree-structured Parzen estimator for intelligent sampling and integrates a pruning mechanism to terminate unpromising trials early. Its computational efficiency is significantly higher than that of the genetic algorithm, with total time reduced by 30% to 40% across different models.

Comprehensive comparison of performance gain and computational cost shows that Optuna is the preferred hyperparameter optimization method for asphalt gel material machine learning studies. It achieves better model performance with less computational resource consumption, and its advantage is more prominent for parameter-sensitive models. Genetic algorithm can also improve model performance but has a slower convergence speed and higher computational cost. It is more suitable for complex models with extremely large parameter spaces. The default parameter configuration can serve as a quick baseline for preliminary exploration, but systematic hyperparameter optimization is necessary to obtain optimal model performance.

### 2.4. Feature Importance Analysis Using SHAP

**Figure 9:**
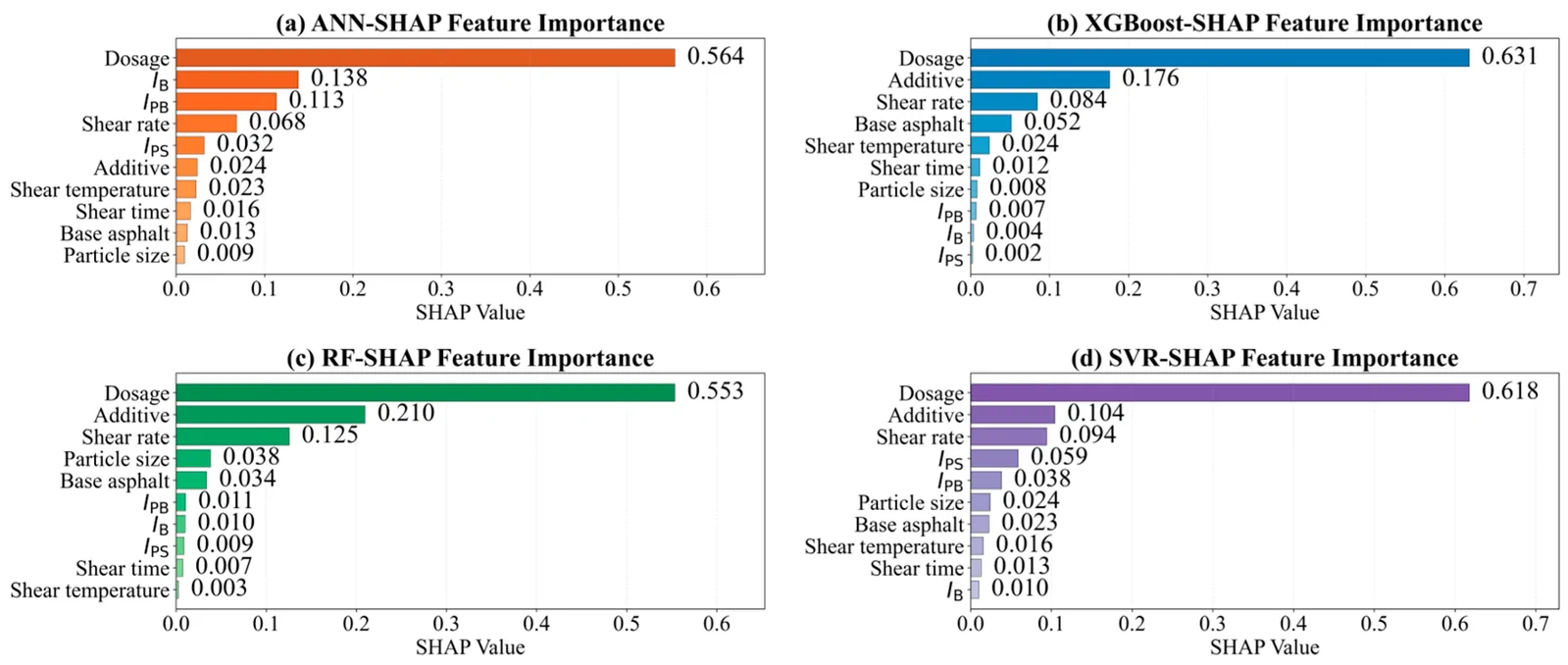
SHAP feature importance comparison for penetration prediction across four models.

**Figure 10:**
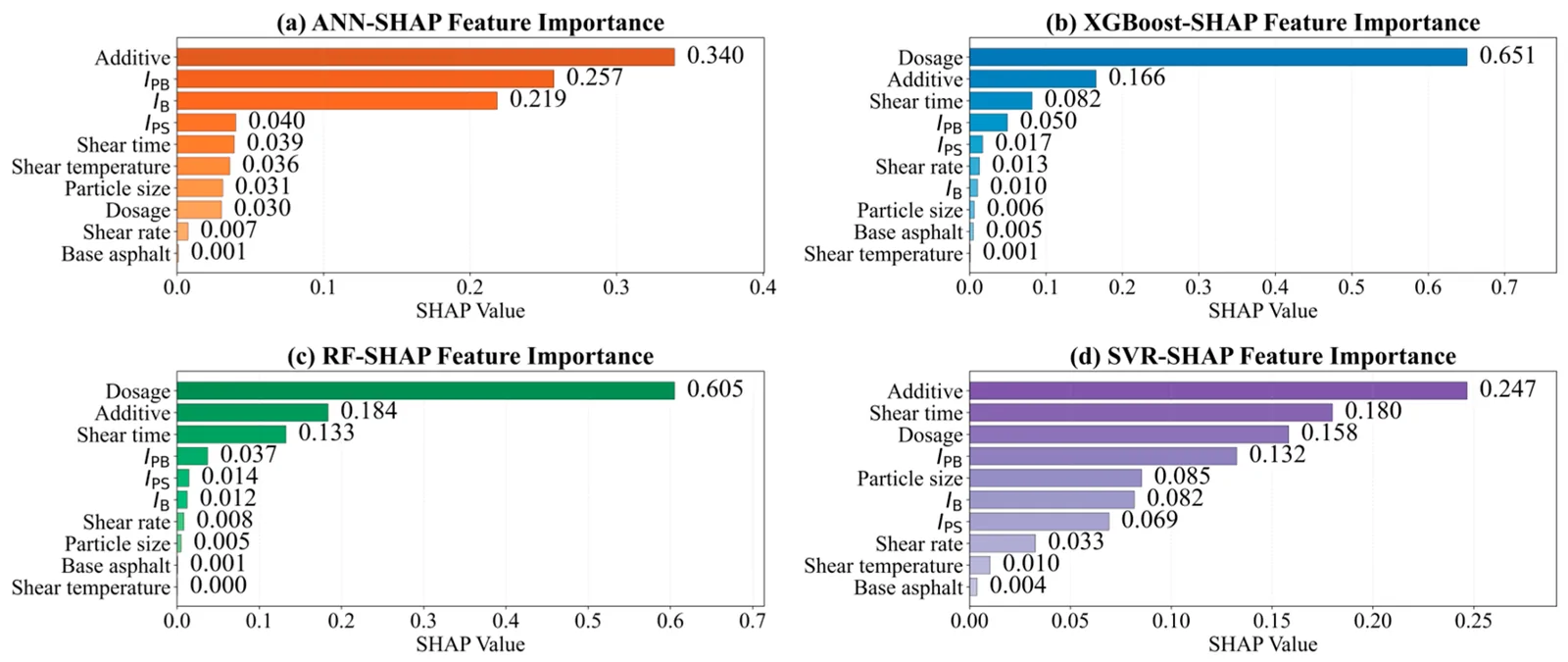
SHAP feature importance comparison for softening point prediction across four models.

**Figure 11:**
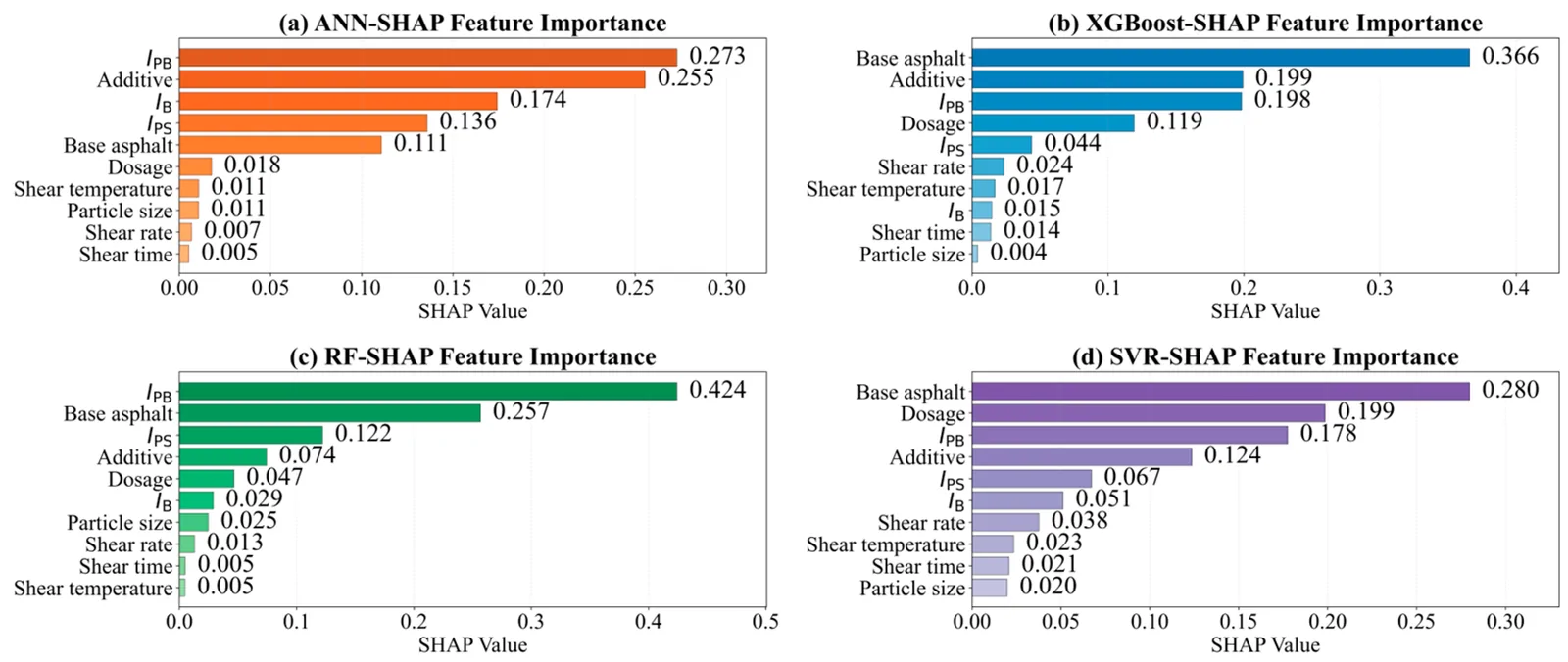
SHAP feature importance comparison for ductility prediction across four models.

**Figure 12:**
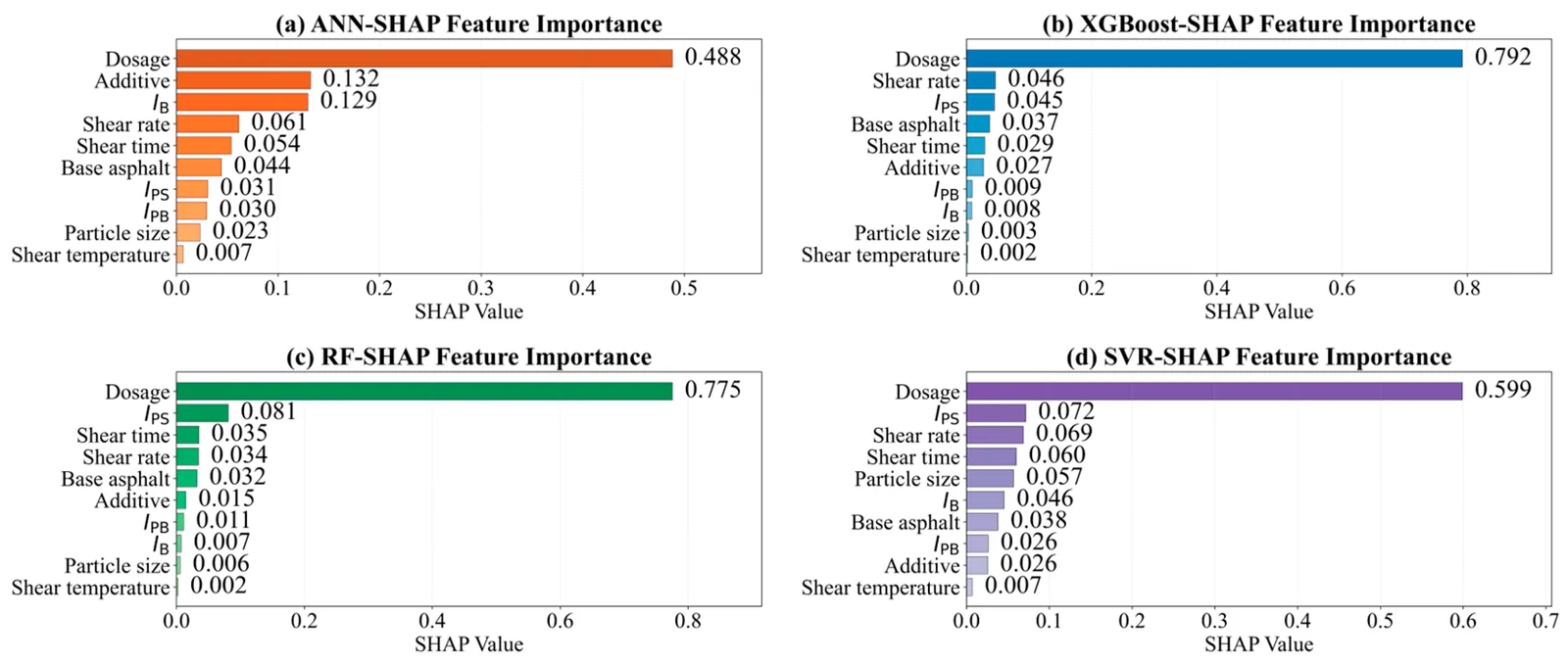
SHAP feature importance comparison for viscosity at 135 °C prediction across four models.

High prediction accuracy alone cannot generate actionable scientific knowledge. This study adopts the SHAP method to quantify the contribution of each input feature to model outputs, and to reveal the core factors governing SBS gel macroscopic performance. Figure 9, Figure 10, Figure 11 and Figure 12 present the feature importance ranking for each of the four target properties, with results from all four models displayed for cross-validation.

The feature importance rankings show high consistency across different models. This consistency confirms that the influence patterns captured by the models reflect real physical mechanisms rather than spurious correlations specific to a single model architecture.

Shear temperature, SBS dosage, and SBS particle size are identified as the three most influential factors across all four performance indicators. Shear temperature controls the viscosity of the asphalt matrix and the mobility of SBS molecular chains. Appropriate temperature promotes sufficient swelling and uniform dispersion of SBS, which is a prerequisite for forming a complete gel network. SBS dosage directly determines the volume fraction of the polymer phase, and is the core factor controlling whether a continuous three-dimensional network can be formed. Particle size directly reflects dispersion quality. Smaller SBS domain size corresponds to more uniform dispersion and better network continuity, which leads to more desirable macroscopic performance.

The relative importance of features varies across different performance indicators. For high-temperature properties including softening point and viscosity, SBS dosage ranks as the most influential feature. At elevated temperatures, the asphalt matrix softens and the system performance relies mainly on the elasticity of the SBS network. Higher polymer content strengthens the network structure and directly improves high-temperature performance. For ductility, the polybutadiene index carries higher weight. Polybutadiene segments are the source of flexibility and elongation capacity of the gel network, and their content directly determines low-temperature deformation capability. For penetration, shear temperature shows the strongest influence, as temperature directly affects the swelling degree and thus the overall consistency of the gel system.

**Figure 13:**
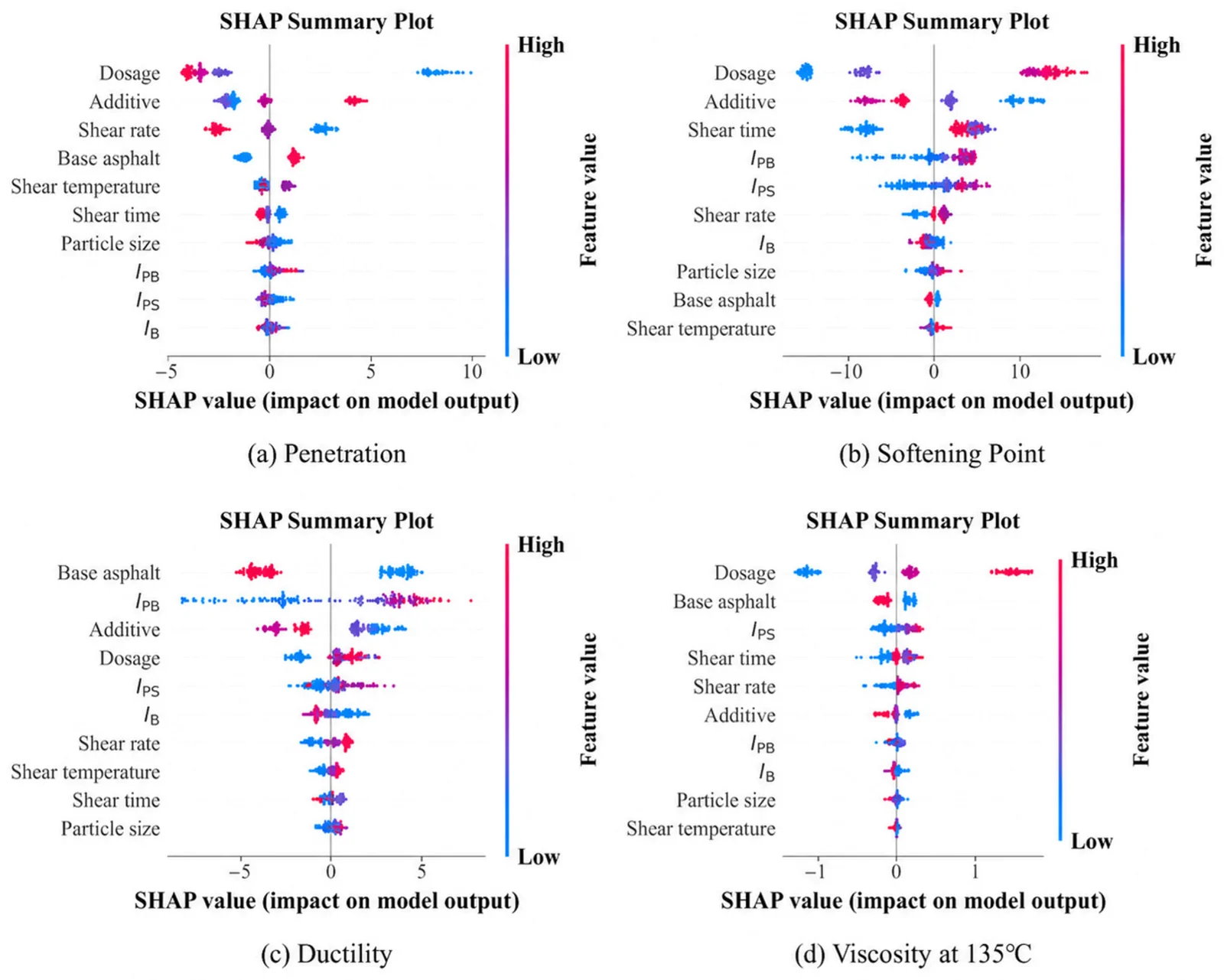
SHAP dependence plots of key features for penetration and softening point prediction.

Figure 13 presents SHAP dependence plots for key features, which visualize how feature values influence prediction outputs across all samples. The clear monotonic trends observed in these plots further verify the stable and regular influence patterns of core factors on gel properties.

**Figure 14:**
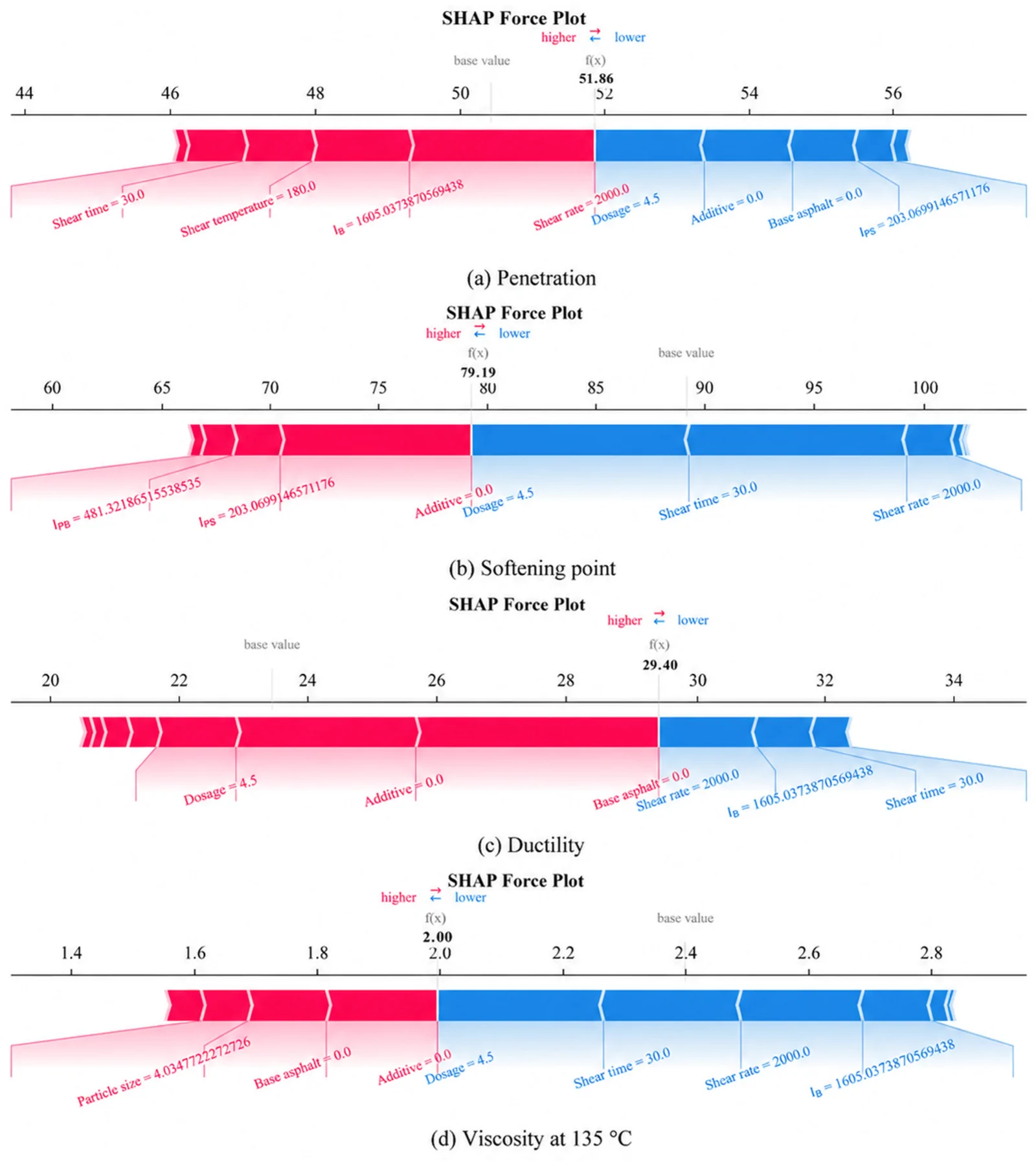
Force plots of key features for penetration and softening point prediction.

Figure 14 displays SHAP force plots for typical samples of four target indicators. The central base value equals the average prediction across all data. Red parts lift predicted performance values, and blue parts reduce predicted values. SBS dosage and polybutadiene index deliver positive effects on penetration and softening point. Longer shear time and higher shear rate form dense gel networks and lower penetration values. Polybutadiene index dominates ductility improvement, while oversized SBS particles reduce low-temperature stretch capacity. Higher SBS dosage raises high-temperature viscosity by increasing internal friction within the gel. The feature action directions match the global importance results from prior SHAP analysis. Force plots decompose single-sample prediction contributions and supply intuitive local interpretations to support the global feature analysis.

The SHAP-derived feature rankings provide quantitative evidence that reinforces the longstanding empirical knowledge in asphalt technology, yet with unprecedented detail. Shear temperature emerges as the dominant factor for penetration, which confirms that the consistency of the final gel is largely determined by the degree of SBS swelling during mixing, a process that is strongly temperature-dependent. SBS dosage controls the volume fraction of the polymer phase, and its top ranking for softening point and viscosity validates the classical percolation theory: only when the dosage exceeds a critical threshold can a continuous three-dimensional network be established, and this network directly elevates the heat resistance and internal friction of the gel. The notable contribution of the polybutadiene index to ductility, especially for low-temperature elongation, is mechanistically sound because the polybutadiene mid-blocks are the sole source of flexibility in the SBS molecule. Interestingly, the FTIR indices carry more weight for ductility than for other properties, suggesting that chemical interactions play a relatively larger role in tensile behavior than in thermal or viscous responses. This differential importance across targets offers a practical guideline: if the goal is to enhance ductility, attention should be paid to preserving the polybutadiene segments from degradation, whereas for high-temperature performance, adjusting the SBS content and shear conditions is more effective. These quantitative insights, which were previously unavailable from conventional experimental designs, demonstrate the added value of combining SHAP with multi-target modeling for intelligent material formulation.

### 2.5. Marginal Effects of Key Features Using Partial Dependence Plots

**Figure 15:**
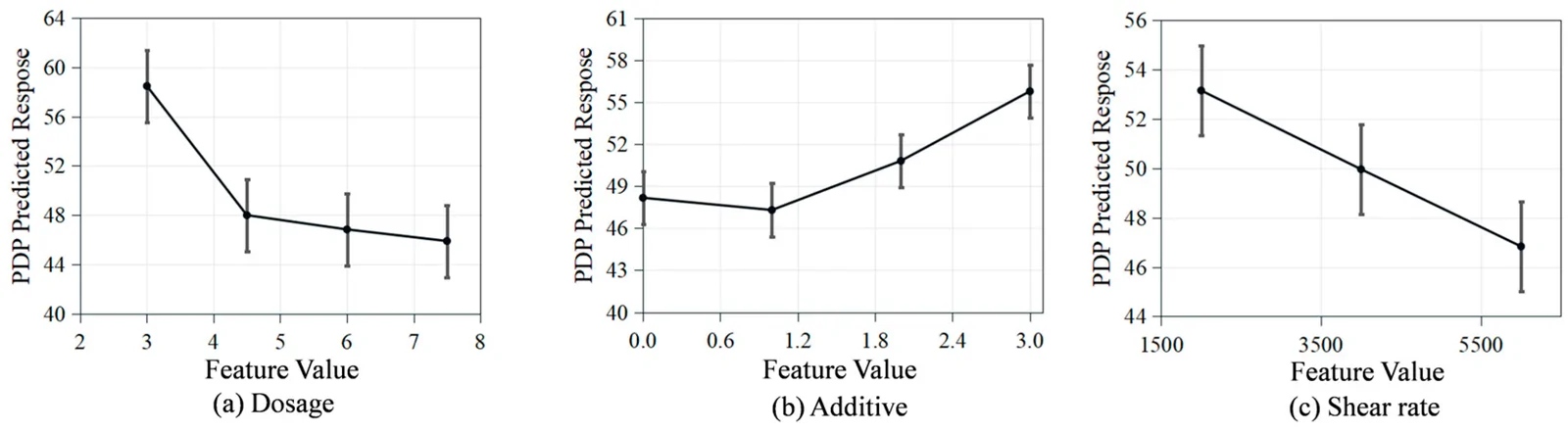
Partial dependence plots of the top three influential features for penetration. Solid lines are partial dependence estimates; shaded bands indicate standard deviation from binning. Scattered points show actual data distribution along each feature axis.

**Figure 16:**
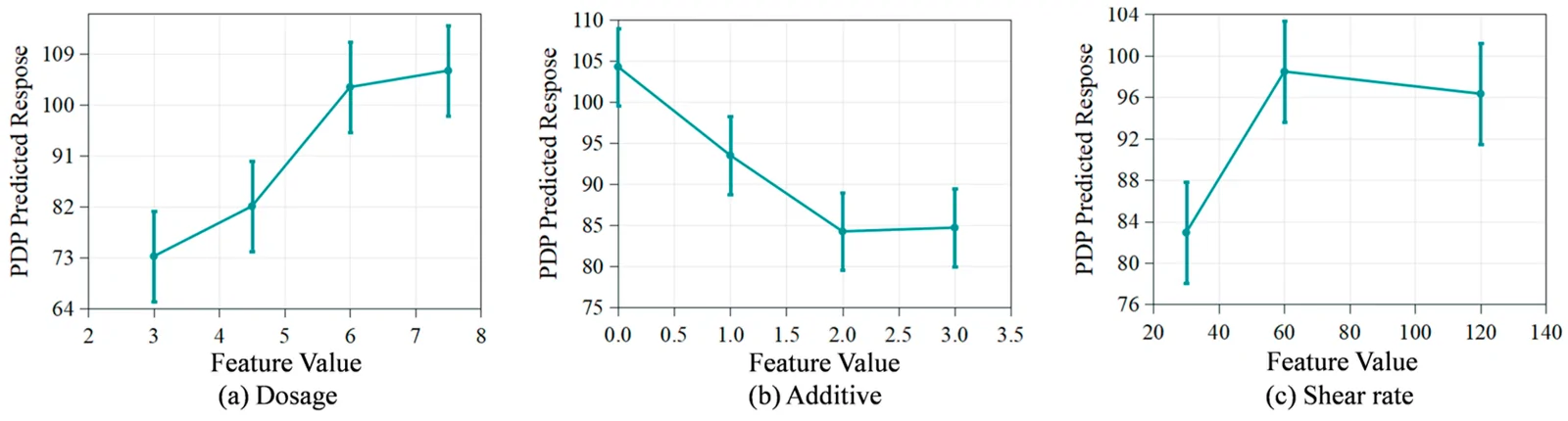
Partial dependence plots of the top three influential features for softening point. Solid lines are partial dependence estimates; shaded bands indicate standard deviation from binning. Scattered points show actual data distribution along each feature axis.

**Figure 17:**
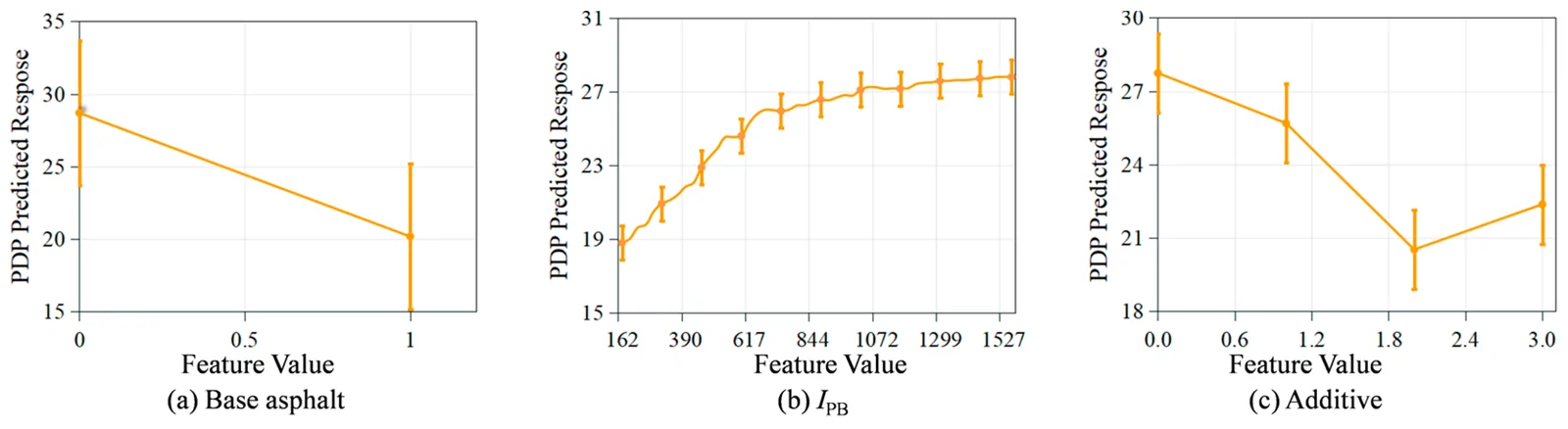
Partial dependence plots of the top three influential features for ductility. Solid lines are partial dependence estimates; shaded bands indicate standard deviation from binning. Scattered points show actual data distribution along each feature axis, with vertical jitter added to avoid overlap. The visible data points confirm data density across the feature range.

**Figure 18:**
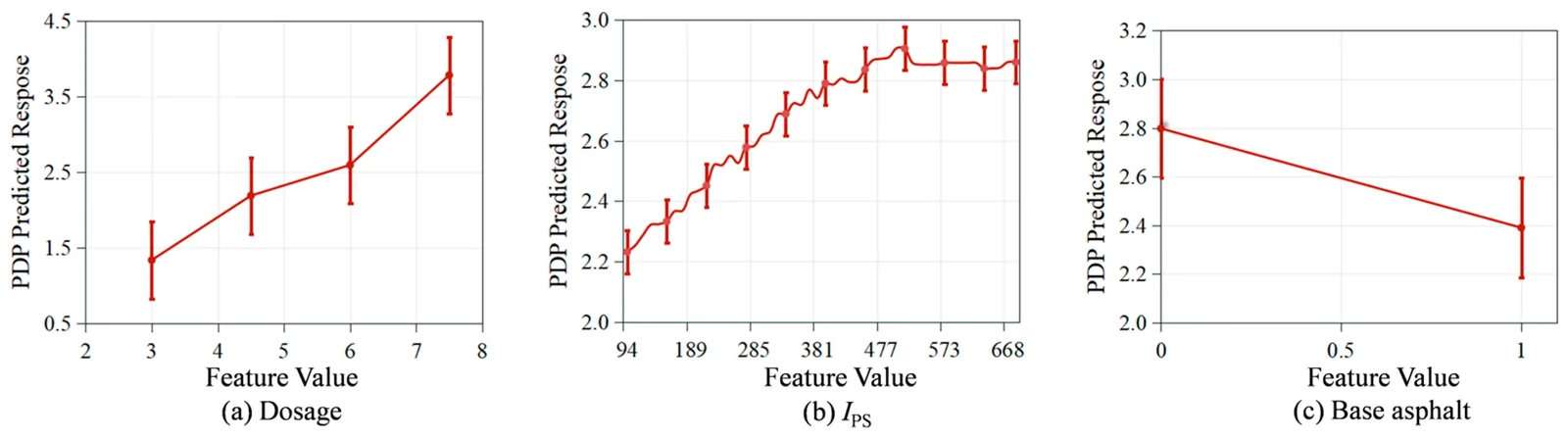
Partial dependence plots of the top three influential features for viscosity at 135 °C. Solid lines are partial dependence estimates; shaded bands indicate standard deviation from binning. Scattered points show actual data distribution along each feature axis.

SHAP analysis identifies the importance ranking of features, while partial dependence plots further reveal the quantitative relationship between individual features and target properties. This method isolates the marginal effect of a single feature by averaging out the influence of all other variables. Figure 15, Figure 16, Figure 17 and Figure 18 present the partial dependence curves of the top three influential features for each target property.

For penetration, Figure 15 shows that penetration decreases continuously with increasing SBS dosage, and the decreasing rate gradually slows down, showing a saturation effect. Penetration drops significantly when dosage increases from 3% to 6%, while the decline becomes less obvious when dosage further increases to 7.5%. This pattern occurs because once the polymer content exceeds the critical network formation threshold, additional SBS brings diminishing marginal improvement to system consistency. Shear rate shows a similar trend, with an inflection point around 4000 rpm. Below this value, increasing shear rate significantly reduces penetration. Beyond this value, further increase in shear intensity brings limited performance improvement, as SBS has already reached a sufficiently dispersed state.

An approximately linear positive relationship between SBS dosage and softening point is illustrated in Figure 16. No obvious saturation plateau appears within the dosage range of this study. This result indicates that increasing SBS content can continuously improve high-temperature performance within the tested range. Shear time exhibits a threshold effect on softening point. Extending shear time significantly improves softening point before 60 min, while the curve flattens out after this point. The 60 min duration corresponds to the critical time required for full swelling and dispersion of SBS under the tested conditions. Further extension of shear time brings limited improvement to network structure.

Figure 17 shows that the polybutadiene index has a strong positive correlation with ductility. Higher polybutadiene content corresponds to better low-temperature elongation capacity, which is fully consistent with theoretical expectations. The base asphalt type also has a significant influence on ductility. Asphalts from different crude oil sources have different component distributions, which lead to different levels of compatibility with SBS and further affect network uniformity and low-temperature performance.

For viscosity at 135 °C, Figure 18 shows that SBS dosage has the most significant influence. Viscosity rises rapidly with increasing SBS content, which imposes negative effects on construction workability. This finding indicates the necessity of balancing high-temperature performance and construction viscosity in practical engineering. The polystyrene index also has a notable positive effect on viscosity. Polystyrene domains act as physical crosslinking points in the gel network. Higher polystyrene content increases the internal friction of the system and leads to higher viscosity. These quantitative marginal effect curves provide direct guidance for material optimization design. Identifying critical points and saturation thresholds helps to maximize process efficiency and reduce production costs while meeting performance requirements.

The partial dependence plots not only reveal the shapes of the relationships but also allow us to define actionable process windows. The saturation of shear temperature around 180 °C and shear time around 60 min indicates that beyond these points, additional energy input yields diminishing returns in performance gain. This is economically significant because prolonged high-shear mixing increases production costs and may even cause thermal degradation of the polymer. Similarly, the dosage-response curve for SBS shows a steep increase from 3% to 6%, followed by a plateau; hence, 6% can be recommended as a cost-effective upper bound for most applications, unless extreme high-temperature performance is specifically required. However, the viscosity at 135 °C rises almost linearly with dosage, which adversely affects workability, and this is a classic trade-off that must be balanced. The curves quantify this trade-off numerically, enabling engineers to select dosages that satisfy both performance and constructability criteria. Furthermore, the effect of shear rate on penetration exhibits a turning point near 4000 rpm, implying that moderate shear is sufficient for achieving adequate dispersion; higher speeds may only introduce unnecessary stress that could break polymer chains. These threshold values, derived purely from data-driven modeling, are consistent with empirical rules but are now backed by statistical confidence. They can be directly incorporated into quality control protocols and production specifications, bridging the gap between machine learning outputs and industrial practice.

### 2.6. Physical Consistency and Knowledge Discovery

Data-driven machine learning models must pass physical consistency verification to ensure scientific validity and generalizability of conclusions. This study verifies the physical consistency of the proposed framework from three dimensions.

The first dimension is inter-target correlation consistency. The four performance indicators predicted by the model maintain the same correlation patterns as those in the measured data. No physically contradictory predictions are observed. For any given set of input parameters, an increase in predicted softening point is always accompanied by a decrease in predicted penetration and an increase in predicted viscosity. This internal consistency confirms that the model has learned the inherent law of gel network evolution rather than fitting isolated data points.

The second dimension is feature effect direction consistency. The influence direction of all key features on performance is fully consistent with established experimental observations and theoretical understanding of polymer gels. No anomalous or counterintuitive influence trends are detected. This consistency rules out the risk that the model captures spurious correlations in the dataset and confirms that the learned patterns reflect real physical mechanisms.

The third dimension is residual randomness. Analysis of model prediction residuals shows that residuals follow a random normal distribution without systematic bias. No significant correlation is observed between residuals and any input feature. This random distribution indicates that the model has fully extracted valid information from the data, and the remaining errors mainly come from random noise in experimental measurements.

Beyond consistency verification, this work also extracts generalizable scientific knowledge from the data-driven model. The study quantitatively validates the gel network theory of SBS-modified asphalt and transforms qualitative mechanism descriptions into quantitative influence weights and marginal effect curves. The three core influencing factors and their critical values identified in this work can be directly applied to guide process parameter optimization in industrial production. For example, controlling shear temperature at around 180 degrees Celsius and shear time around 60 min can produce qualified modified asphalt with relatively low energy consumption. In addition, the confirmed strong correlation among multiple performance indicators makes it possible to infer hard-to-measure properties from easy-to-measure indicators, which reduces testing cost and accelerates formulation screening.

The multi-scale machine learning framework established in this study realizes a full-chain quantitative mapping from process and composition to microstructure and then to macroscopic performance. This research paradigm combining materials genome database, explainable artificial intelligence, and multi-target correlation analysis can also be extended to the development of other polymer gel materials.

## 3. Conclusions

This study constructs a multi-scale machine learning framework for SBS-modified asphalt gels, integrating processing parameters, material composition features, and microstructural gene indicators to achieve accurate prediction and interpretable analysis of four macroscopic properties. The main conclusions are as follows.

The dataset compiled from the gene database and supplementary experiments contains 1072 valid samples covering a broad parameter space, providing sufficient data support for reliable machine learning modeling. Multi-target correlation analysis reveals strong correlations among the four performance indicators. The Pearson correlation coefficient between penetration and softening point is −0.681, between penetration and viscosity is −0.704, and between softening point and viscosity is 0.804. All properties share a common physical basis corresponding to the integrity of the SBS gel network. The first principal component explains over 72% of the total variance, providing a physical foundation for multi-target collaborative material design.

All four machine learning models achieve high prediction accuracy. The support vector machine with radial basis function kernel attains R^2^ values of 0.9997 for penetration and 0.9996 for ductility. The artificial neural network reaches 0.9996 for softening point, and extreme gradient boosting achieves 0.9993 for viscosity at 135 °C. These results verify the advantage of kernel methods for medium-scale polymer gel datasets with smooth nonlinear relationships. Optuna-based optimization improves the average R^2^ by 2.1% over default configurations and reduces computational time by 30% to 40% compared with genetic algorithm optimization, establishing it as the preferred optimization scheme for asphalt material machine learning studies.

SHAP interpretability analysis quantitatively identifies shear temperature, SBS dosage, and SBS particle size as the three core factors governing the macroscopic performance of SBS asphalt gels. Partial dependence plots further reveal the marginal effect curves and critical thresholds of each factor. These findings are fully consistent with polymer gel theory and provide quantitative design guidelines that can directly inform material formulation and process optimization.

The proposed multi-scale, interpretable, multi-target-correlated framework bypasses the traditional trial-and-error black-box mode of asphalt material development and provides data-driven technical support for intelligent design of SBS-modified asphalt gels. Future work can expand the dataset to include more modifier types and aging conditions to broaden the model’s applicability and can introduce physically constrained machine learning methods to further enhance physical interpretability and extrapolation capability.

## 4. Materials and Methods

### 4.1. Materials and Dataset

**Figure 19:**
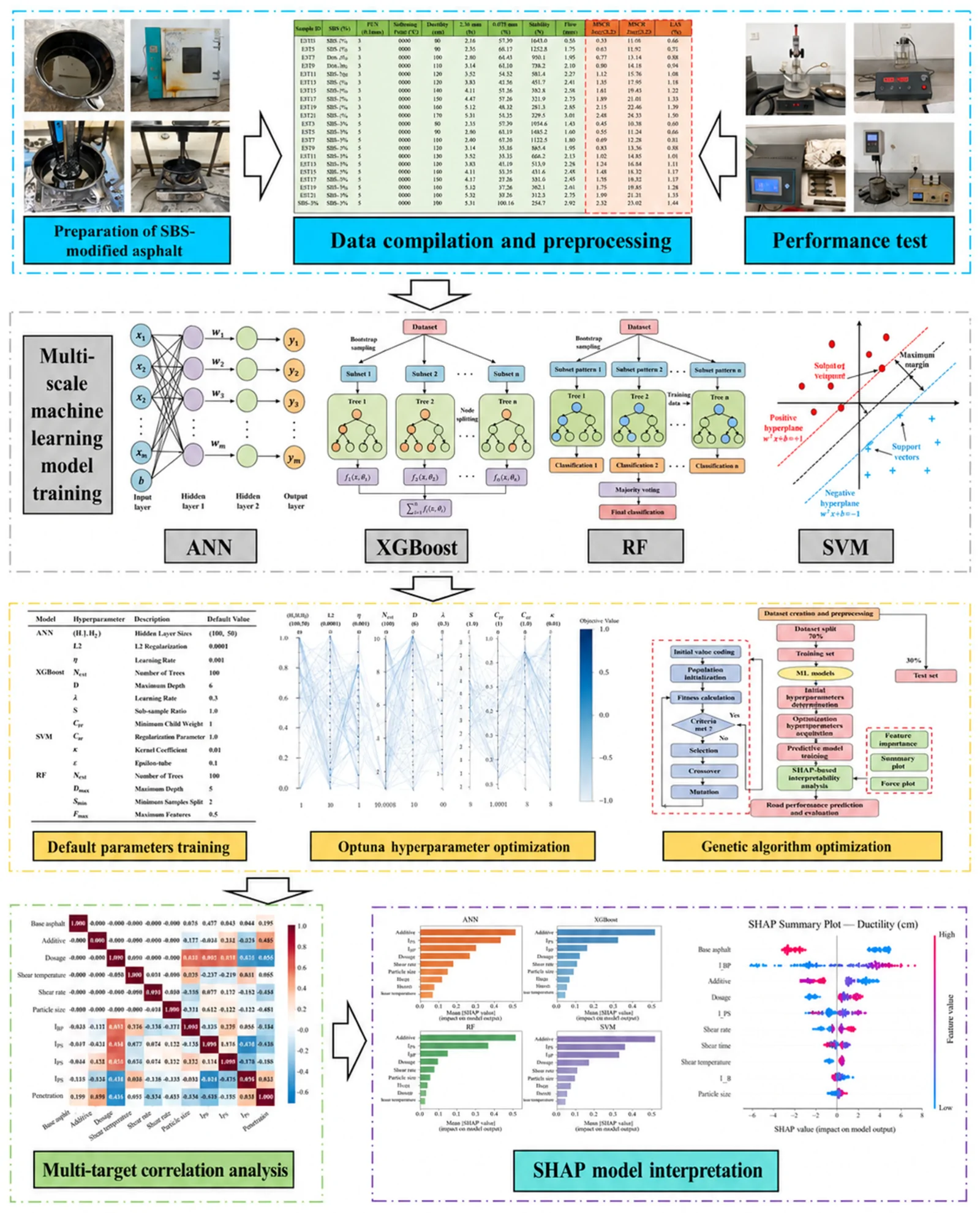
Overall workflow comprising five stages: data compilation and preprocessing, multi-target correlation analysis, model training with default parameters, hyperparameter optimization using three strategies (default, GA, and Optuna), and model interpretation via SHAP and partial dependence plots.

As illustrated in Figure 19, the overall research workflow comprised five sequential stages. The first stage was data compilation and preprocessing. This involved extracting data from the gene database and supplementary experiments, cleaning the data, encoding categorical features, and standardizing the continuous features. The second stage was multi-target correlation analysis. This involved calculating Pearson correlation coefficients and performing principal component analysis to reveal the relationships among the four target properties. The third stage was model development. This involved training the four machine learning models with default parameters. The fourth stage was hyperparameter optimization. This involved optimizing the models using Optuna, genetic algorithm, and default configurations, and comparing their performance. The fifth stage was model interpretation. This involved using SHAP and partial dependence plots to extract scientific insights from the optimized models.

The dataset used in this study was assembled from two complementary sources. The primary source is the Southwest Jiaotong University asphalt/cement‑based materials gene‑performance quantitative structure‑property relationship dataset, accessible at https://nbsdc.cn/general/dataDetail?id=69d7cd46f175606608ea1e0f&type=1 (accessed on 15 July 2026). Conceived within the materials genome framework, it functions as a multi-scale and multi-dimensional data integration platform for asphalt-based materials comprising 864 samples. The database encompasses chemical composition, microstructural characteristics, and macroscopic performance across multiple scales. It provides a foundational data resource for constitutive relationship analysis, performance prediction, and intelligent design of asphalt-based materials. The secondary source comprised 208 samples from supplementary laboratory experiments conducted by the research group of the authors. These experiments were designed to fill data gaps and ensure comprehensive coverage of the processing parameter space. All experiments followed standardized protocols to guarantee data consistency across sources.

**Figure 20:**
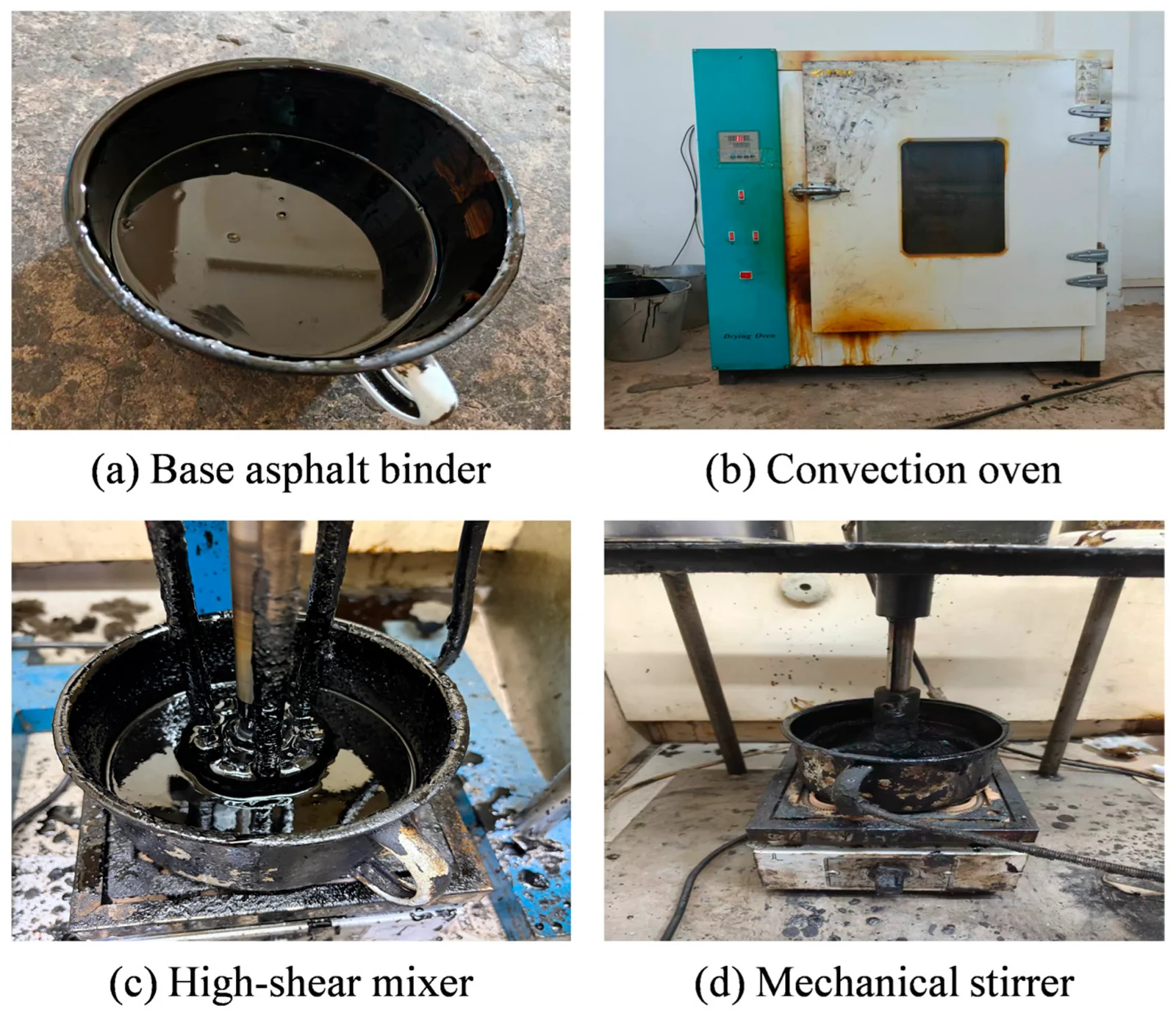
Schematic illustration of the SBS-modified asphalt binder preparation process: (**a**) base asphalt binder; (**b**) convection oven for pre-heating and SBS swelling; (**c**) high-shear mixer for SBS dispersion; (**d**) mechanical stirrer for post-blending.

The full preparation workflow of SBS-modified asphalt binders in supplementary laboratory experiments made by the authors is displayed in Figure 20. Raw base asphalt binder is prepared as the initial raw material. A convection oven provides stable thermal environment to complete asphalt pre-heating and SBS particle swelling treatment. A high-shear mixer applies intense mechanical shear force to achieve uniform dispersion of SBS modifier within asphalt matrix. A mechanical stirrer carries out subsequent low-speed blending to homogenize the overall gel system after high shear processing.

The input feature set comprised 10 variables categorized into three groups. Table 3 and Table 4 present the overview of sample features and output variables of the dataset. The first group consisted of three categorical composition features. Base asphalt type had two levels: ES70 and SK70. SBS type had four levels: SBS-796, SBS-791H, SBS-792, and SBS-161B. SBS dosage had four levels: 3%, 4.5%, 6%, and 7.5% by weight of base asphalt. These composition features define the material system before processing and fundamentally influence the gel network formation.

The second group consisted of three continuous processing parameters. Shear temperature was varied at three levels: 170 degrees Celsius, 180 degrees Celsius, and 190 degrees Celsius. Shear rate was varied at three levels: 2000 rpm, 4000 rpm, and 6000 rpm. Shear time was varied at three levels: 30 min, 60 min, and 120 min. These three parameters define the processing conditions under which SBS is dispersed into the asphalt matrix and the gel network is formed. The combination of composition features and processing parameters determines the initial state and the kinetic conditions for gel network development.

The third group consisted of four continuous microstructural genes. Particle size represented the average diameter of SBS domains as determined from fluorescence microscopy images, measured in micrometers. $I_{PS}$ denoted the polystyrene index calculated from Fourier transform infrared spectroscopy as the ratio of the absorbance at 699 reciprocal centimeters to the absorbance at 1376 reciprocal centimeters. This index reflects the relative content of polystyrene segments in the gel network. $I_{PB}$ denoted the polybutadiene index calculated as the ratio of the absorbance at 966 reciprocal centimeters to the absorbance at 1376 reciprocal centimeters. This index indicates the relative content of polybutadiene segments. $I_{B}$ denoted the butadiene index calculated as the ratio of the absorbance at 911 reciprocal centimeters to the absorbance at 1376 reciprocal centimeters. These four microstructural genes provide quantitative measures of the SBS dispersion state and the chemical integrity of the polymer network within the asphalt gel matrix.

The target variables comprised four macroscopic performance indicators that are conventionally used to characterize SBS-modified asphalt. Penetration was measured in 0.1 mm at 25 degrees Celsius. It indicates the consistency or softness of the asphalt gel. Softening point was measured in degrees Celsius. It indicates the high-temperature performance and the thermal stability of the gel network. Ductility was measured in centimeters at 25 degrees Celsius. It indicates the low-temperature deformation capacity of the gel. Viscosity at 135 degrees Celsius was measured in pascal-seconds. It indicates the workability and processing characteristics of the gel at elevated temperatures.

**Figure 21:**
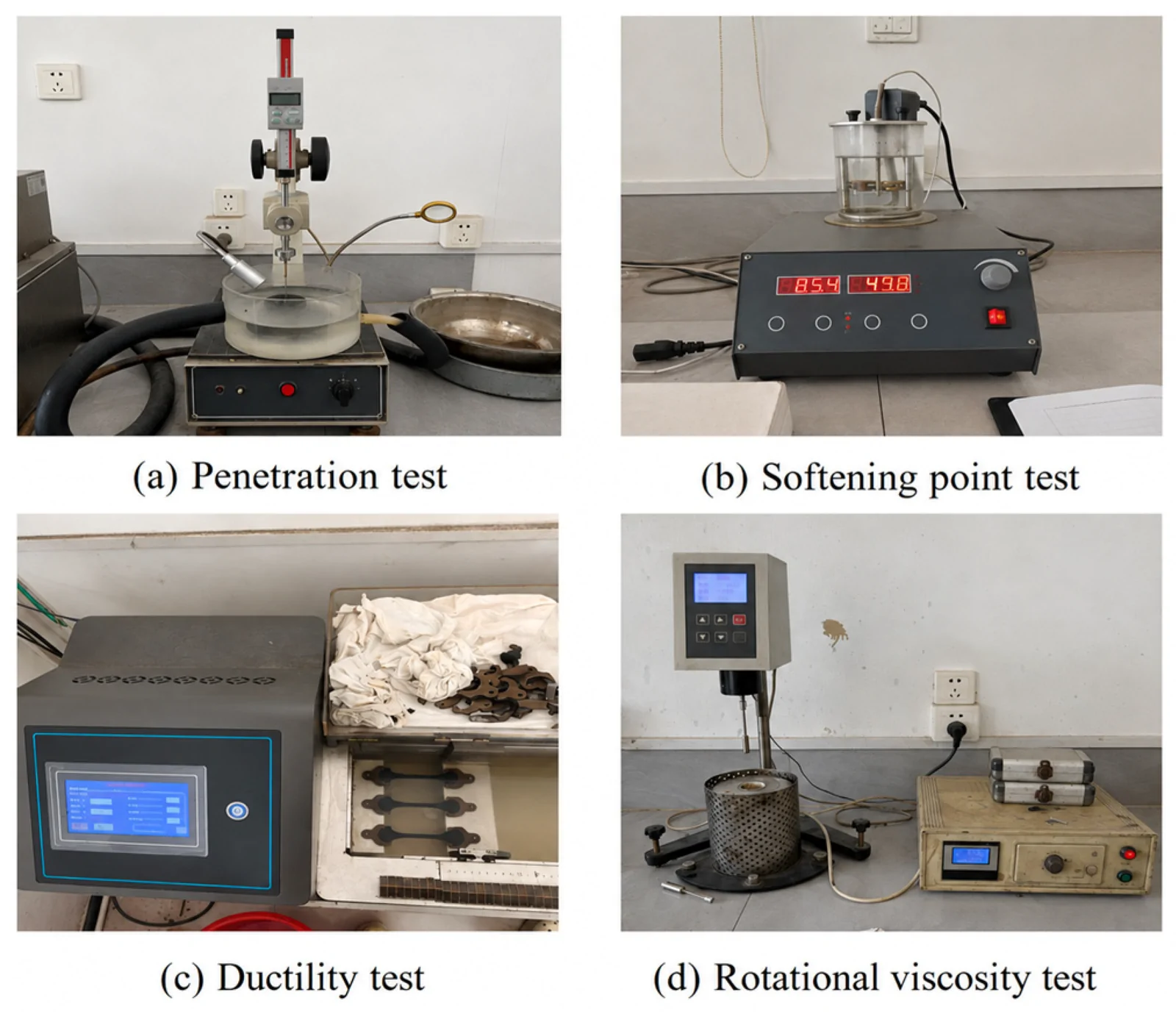
Macroscopic performance characterization of SBS-modified asphalt binder: (**a**) penetration test at 25 °C; (**b**) softening point test; (**c**) ductility test at 25 °C; (**d**) rotational viscosity test at 135 °C.

This study adopts standard asphalt material test equipment to complete all four macroscopic performance tests for supplementary experimental samples. The whole set of performance characterization devices is presented in Figure 21. A penetration test apparatus (Model LYY‑10A, Wuxi Petroleum Instrument Co., Ltd., Wuxi, China) completes penetration index measurement under constant 25 °C temperature condition. A digital softening point tester (Model WSY‑025B, Wuxi Petroleum Instrument Co., Ltd., Wuxi, China) measures the softening point value following standard test specifications. A ductility testing machine (Model LYY‑10‑1, Wuxi Petroleum Instrument Co., Ltd., Wuxi, China) records the ultimate stretching length of asphalt samples at 25 °C to quantify low‑temperature ductility performance. A rotational viscosity instrument (Model NDJ‑1C, Wuxi Petroleum Instrument Co., Ltd., Wuxi, China) acquires the high‑temperature viscosity data of modified asphalt under 135 °C constant heating condition.

After data cleaning, which involved the removal of outliers and incomplete records, the final dataset comprised 1072 valid samples. The following factors were systematically varied across the samples: base asphalt type, SBS type, SBS dosage, shear temperature, shear rate, and shear time. The full factorial design with these six factors at their respective levels would have produced an excessive number of combinations. Therefore, a strategically designed subset was selected to ensure comprehensive coverage of the parameter space while maintaining experimental feasibility. This design ensured that the dataset captured the essential variations in processing conditions and material composition relevant to SBS-modified asphalt gel formation. Table 5 presents the statistical summary of all continuous features and targets. For the categorical features, the distribution is as follows: base asphalt type ES70 accounts for 50% of the samples and SK70 accounts for the other 50%; SBS type 796 accounts for 25% of the samples, SBS type 791H accounts for 25%, SBS type 792 accounts for 25%, and SBS type 161B accounts for 25%; SBS dosage is distributed as 25% at 3%, 25% at 4.5%, 25% at 6%, and 25% at 7.5%.

### 4.2. Multi-Target Performance Correlation Analysis

Prior to machine learning modeling, a systematic correlation analysis was conducted to reveal the inherent physical relationships among the four target properties. This analysis served two purposes. The first purpose was to validate the physical consistency of the dataset by examining whether the correlations among properties align with established physicochemical principles of SBS-modified asphalt gels. The second purpose was to provide insights into the common underlying mechanisms governing these properties, namely the integrity of the SBS gel network.

Pearson correlation coefficients were calculated to quantify the linear relationships between all pairs of target variables. The Pearson correlation coefficient r between two variables X and Y is defined as:

(1) $$r=\frac{\sum_{i=1}^{n}\left(X_{i}-X)(Y_{i}-Y\right)}{\sqrt{\sum_{i=1}^{n}{\left(X_{i}-\bar{X}\right)}^{2}\sum_{i=1}^{n}{\left(Y_{i}-\bar{Y}\right)}^{2}}}$$

where n is the number of samples, $X_{i}$ and $Y_{i}$ are the individual sample values, and $\bar{X}$ and $\bar{Y}$ are the mean values of X and Y, respectively. The coefficient ranges from −1 to +1, where +1 indicates a perfect positive linear relationship, −1 indicates a perfect negative linear relationship, and 0 indicates no linear relationship. A correlation matrix was constructed and visualized as a heatmap.

### 4.3. Data Preprocessing

Before model training, all continuous input features were standardized to have zero mean and unit variance. Standardization is essential for machine learning models that are sensitive to feature scales, particularly support vector machine and artificial neural network. The standardization was performed using the following transformation:

(2) $$z=\frac{x-\mu }{\sigma }$$

where x is the original feature value, μ is the mean of the feature, and σ is the standard deviation of the feature. This transformation ensures that each continuous feature contributes equally to the model training process. It prevents features with larger magnitudes from dominating the learning algorithm. The standardization parameters were calculated from the training set and applied to both the training and test sets to prevent data leakage.

For the categorical features, one-hot encoding was applied. Base asphalt type was encoded as two binary variables: ES70 and SK70. SBS type was encoded as four binary variables: SBS-796, SBS-791H, SBS-792, and SBS-161B. SBS dosage was encoded as four binary variables representing the four dosage levels. One-hot encoding transforms categorical variables into a format that can be provided to machine learning algorithms. Each category is represented as a binary vector where only one element is 1 and the others are 0. This encoding preserves the categorical nature of the features without imposing any ordinal relationship.

### 4.4. Machine Learning Models

Four machine learning models with fundamentally different architectural principles were selected to capture the complex nonlinear relationships between input genes and target properties.

#### 4.4.1. Subsubsection

The support vector machine with RBF kernel was implemented using the scikit-learn library (Version 1.2.0, INRIA, Paris, France) [45]. SVR maps input features into a high-dimensional space via the kernel function and performs linear regression in that space, tolerating deviations up to ε. The RBF kernel is defined in Equation (3). The kernel coefficient gamma controls the influence range of a single training sample: larger gamma values produce more complex decision boundaries with local influence, while smaller gamma values yield smoother boundaries.

The regularization parameter C does not enter the kernel calculation directly but appears in the SVR optimization objective. The primal optimization problem is:

(3) $$\begin{array}{c} {min}_{w,\;b,\;\xi_{i},\;\xi_{i}^{*}}\frac{1}{2}{\left\|w\right\|}^{2}+C\sum_{i=1}^{n}\left(\xi_{i}+\xi_{i}^{*}\right)\\ \mathrm{subject}\;\mathrm{to}:\;\;\;\;\;\;\;\;\;\;\;\;\;\;\;\;\;\;\;\;\;\;\;\;\;\\ y_{i}-(w\cdot \phi (x_{i})+b)\leq \varepsilon +\xi_{i}\\ w\cdot \phi (x_{i})+b-y_{i}\leq \varepsilon +\xi_{i}^{*}\\ \xi_{i},\;\xi_{i}^{*}\geq 0 \end{array}$$

where w and b are the weight vector and bias, $\phi (x_{i})$ is the mapping of input $x_{i}$ into the high-dimensional space, $\xi_{i}$ and $\xi_{i}^{*}$ are slack variables, and ε is the width of the insensitive tube. C penalizes deviations exceeding ε. A large C forces the model to fit training data more strictly at the risk of overfitting, whereas a small C allows larger training errors but yields a smoother regression function. Thus, C and gamma influence model performance from different aspects. C controls regularization strength, while gamma determines the local influence of the kernel. Both must be tuned jointly to achieve optimal generalization.

#### 4.4.2. Artificial Neural Network

The artificial neural network was implemented as a feedforward neural network using the scikit-learn library (Version 1.2.0, INRIA, Paris, France) [46]. The network architecture consisted of an input layer with neurons corresponding to the encoded input features, two hidden layers with ReLU activation functions, and an output layer with one neuron for single-target prediction. This activation function introduces nonlinearity while mitigating the vanishing gradient problem. The number of neurons in each hidden layer, the learning rate for weight updates, and the regularization parameter alpha were treated as hyperparameters to be optimized. The artificial neural network is capable of approximating any continuous function given sufficient hidden units and is well-suited for modeling complex nonlinear relationships.

#### 4.4.3. Random Forest

The random forest was implemented as an ensemble of decision trees using the scikit-learn library (Version 1.2.0, INRIA, Paris, France) [47]. Random forest builds multiple decision trees on bootstrapped samples of the training data and averages their predictions. This technique is known as bagging. The approach reduces overfitting and improves generalization performance. Each tree in the forest is constructed by recursively partitioning the feature space based on the best split at each node. The random forest captures complex interactions and nonlinearities through the hierarchical structure of decision trees. The key hyperparameters include the number of trees in the forest, the maximum depth of each tree, the minimum number of samples required to split an internal node, and the minimum number of samples required to be at a leaf node.

#### 4.4.4. Extreme Gradient Boosting

The extreme gradient boosting was implemented using the XGBoost library (Version 1.7.0, Distributed Machine Learning Community, Seattle, WA, USA) [48]. Extreme gradient boosting is a gradient boosting framework that sequentially builds decision trees. Each new tree corrects the errors of the previous ensemble. The algorithm uses a regularized objective function that balances the trade-off between bias and variance. This helps to prevent overfitting. The gradient boosting mechanism makes extreme gradient boosting particularly effective for capturing complex patterns in structured data. The key hyperparameters include the learning rate, which controls the contribution of each tree; the number of estimators, which determines the size of the ensemble; the maximum depth of each tree; the subsample ratio, which specifies the fraction of samples used for training each tree; and the column sampling ratio, which specifies the fraction of features used for training each tree.

### 4.5. Hyperparameter Optimization

Hyperparameter optimization is a critical step in machine learning modeling that significantly influences model performance. This study employed three hyperparameter optimization approaches and systematically compared their effectiveness.

#### 4.5.1. Default Parameter Configuration

The default parameter configuration served as the baseline for comparison. The default parameters were those provided by the scikit-learn and XGBoost libraries. These default values are based on empirical settings for general datasets and were not adjusted for the specific characteristics of our dataset. The default configuration represents the simplest approach to model training, requiring no additional computational cost for hyperparameter search. However, default parameters may not be optimal for a specific dataset, potentially leading to suboptimal model performance. The default configurations used in this study were as follows. For the support vector machine, the default C value was 1.0, gamma was set to scale, and epsilon was 0.1. For the artificial neural network, the default hidden layer sizes were 100 neurons per layer, the default learning rate was 0.001, and the default alpha was 0.0001. For the random forest, the default number of trees was 100, the default maximum depth was None, meaning unlimited, and the default minimum samples per split was 2. For extreme gradient boosting, the default learning rate was 0.3, the default number of estimators was 100, and the default maximum depth was 6. These default values are based on the recommended settings in the scikit-learn (version 1.2.0) and XGBoost (version 1.7.0) library documentation, which were originally established for general-purpose datasets. Specifically, the default C value of 1.0 for SVM follows the statistical learning theory framework proposed by Vapnik [45]; the default number of trees for random forest follows Breiman’s original algorithm [47]; and the default learning rate and tree depth for XGBoost follow the gradient boosting recommendations of Chen and Guestrin [48].

#### 4.5.2. Genetic Algorithm Optimization

The genetic algorithm optimization was implemented following the approach reported by Wu et al. in their study on cold recycled mix asphalt performance prediction [31]. A genetic algorithm is a metaheuristic optimization method inspired by the process of natural selection. It operates by maintaining a population of candidate solutions, which evolve toward better solutions over successive generations through selection, crossover, and mutation operations. In the context of hyperparameter optimization, each individual in the population represents a set of hyperparameter values. The fitness of each individual is evaluated by the performance of the corresponding machine learning model.

The genetic algorithm configuration in this study was as follows. The population size was set to 20 individuals. The number of generations was set to 50. The crossover probability was set to 0.8, meaning that 80% of the selected pairs would undergo crossover to produce offspring. The mutation probability was set to 0.1, meaning that each gene had a 10% chance of being mutated. The selection mechanism was tournament selection with a tournament size of 3. The fitness function was the negative $R^{2}$ score obtained from 5-fold cross-validation, with the optimization objective being the maximization of $R^{2}$. This configuration was consistent with the settings commonly reported in asphalt materials machine learning studies.

#### 4.5.3. Optuna Optimization

Optuna (Version 3.4.0, Preferred Networks, Inc., Tokyo, Japan) is a hyperparameter optimization framework that uses a tree-structured Parzen estimator as the sampling algorithm. Unlike grid search or random search, Optuna intelligently samples the hyperparameter space based on the history of previous evaluations. This achieves better results with fewer iterations. The tree-structured Parzen estimator models the distribution of hyperparameters separately for good and bad trials. It uses this model to suggest promising hyperparameter values for subsequent trials. Additionally, Optuna incorporates a pruning algorithm that can terminate unpromising trials early, significantly improving optimization efficiency. For each model, the hyperparameter search spaces for the Optuna optimization process were configured as shown in Table 6.

#### 4.5.4. Comparison of Optimization Methods

The three hyperparameter optimization methods were systematically compared using the following criteria. The first criterion was the predictive performance, measured by the mean *R*^2^, mean RMSE, and mean MAE from 5-fold cross-validation. The second criterion was the stability of the optimization, measured by the standard deviation of the performance metrics across the five folds. The third criterion was the computational efficiency, measured by the total time required for the optimization process. This comprehensive comparison aimed to provide guidance for selecting appropriate hyperparameter optimization methods in future asphalt materials machine learning studies.

### 4.6. Model Evaluation

The models were evaluated using 5-fold cross-validation to ensure robustness and prevent overfitting. In 5-fold cross-validation, the dataset is randomly partitioned into five equal-sized subsets. The model is trained on four subsets and tested on the remaining subset. This process is repeated five times with each subset serving as the test set once. The performance metrics are then averaged over the five folds. This provides a more reliable estimate of model generalization performance than a single train-test split.

Three evaluation metrics were employed. The coefficient of determination *R*^2^ measures the proportion of variance in the target variable that is explained by the model. *R*^2^ is defined as:

(4) $$R^{2}=1-\frac{\sum_{i=1}^{n}(y_{i}-{\hat{y}}_{i}{)}^{2}}{\sum_{i=1}^{n}(y_{i}-\bar{y}{)}^{2}}$$

where $y_{i}$ is the actual value, ${\hat{y}}_{i}$ is the predicted value, and $\bar{y}$ is the mean of the actual values. *R*^2^ ranges from 0 to 1, with higher values indicating better predictive performance. The RMSE measures the standard deviation of the prediction errors. RMSE is defined as:

(5) $$RMSE=\sqrt{\frac{1}{n}\sum_{i=1}^{n}{\left(y_{i}-{\hat{y}}_{i}\right)}^{2}}$$

Lower RMSE values indicate better accuracy. The mean absolute error measures the average absolute deviation between the predicted and actual values. MAE is defined as:

(6) $$MAE=\frac{1}{n}\sum_{i=1}^{n}\left|y_{i}-{\hat{y}}_{i}\right|$$

Lower MAE values indicate better accuracy. These three metrics provide complementary information about model performance. *R*^2^ indicates the explanatory power of the model. RMSE is sensitive to large errors. MAE provides a more intuitive measure of average prediction error. The performance of the four models was systematically compared using the cross-validation mean scores and standard deviations. Pairwise statistical tests were conducted to determine whether the performance differences between models were statistically significant.

### 4.7. SHAP Model Interpretability

Model interpretability was achieved through two complementary techniques: SHAP and partial dependence plots. SHAP is a game-theoretic approach to explaining the output of machine learning models. The SHAP value for a feature represents the average contribution of that feature to the prediction, computed across all possible coalitions of features. The SHAP value $\phi_{i}$ for feature i is defined as:

(7) $$\phi_{i}=\sum_{S\subseteq F\backslash \{i\}}\frac{\left|S\right|!(\left|F\right|-\left|S\right|-1)!}{\left|F\right|!}[f(S\cup \{i\})-f(S)]$$

where F is the set of all features, S is a subset of features excluding feature i, f(S) is the prediction of the model with features in subset S, and the sum is over all possible subsets S. This formulation ensures that the SHAP values satisfy local accuracy, consistency, and missingness properties.

SHAP provides both global and local interpretability. For global interpretability, SHAP summary plots were generated to rank feature importance across all samples and to visualize the direction and magnitude of each feature’s impact on predictions. The summary plot combines feature importance with the sign of the effect, showing which features have positive or negative contributions to the predicted value. For local interpretability, SHAP waterfall plots were generated for representative samples to illustrate how individual predictions are composed of feature contributions. The waterfall plot shows the base value, which is the average prediction, and how each feature shifts the prediction toward the final output.

Partial dependence plots were used to visualize the marginal effect of individual features on the predicted target properties, independent of other features. The partial dependence plot shows how the predicted value changes as a function of a single feature while averaging out the effects of all other features. For each key feature identified by SHAP, partial dependence plots were generated to reveal the shape of the relationship, which could be linear, nonlinear, threshold, or U-shaped; the optimal range for maximizing or minimizing specific properties; and potential interaction effects visualized through two-dimensional partial dependence plots for feature pairs. The combination of SHAP and partial dependence plots enables a comprehensive understanding of the gene–performance relationships, transforming the machine learning model from a black-box predictor into a tool for scientific discovery.

## Figures and Tables

**Table 1:** Overall performance comparison of four machine learning models.

Performance	ML Model	R^2^	RMSE	MAE
Penetration	ANN	0.9687	1.681	1.32
	XGBoost	0.9997	0.164	0.12
	RF	0.9996	0.276	0.21
	SVM	0.9955	0.926	0.72
Softening point	ANN	0.9980	0.617	0.48
	XGBoost	0.9839	1.751	1.37
	RF	0.9974	0.500	0.39
	SVM	0.9954	0.664	0.52
Ductility	ANN	0.9716	1.651	1.29
	XGBoost	0.9996	0.196	0.15
	RF	0.9673	0.170	0.13
	SVM	0.9993	0.025	0.020
Viscosity at 135 °C	ANN	0.9915	0.087	0.068
	XGBoost	0.9952	0.065	0.051
	RF	0.9687	1.681	1.32
	SVM	0.9997	0.164	0.12

**Table 2:** Computational cost comparison of three hyperparameter optimization methods reported in seconds. Values for default configuration are single-training times without tuning; GA and Optuna values include the total optimization time with five-fold cross-validation.

Computational Cost (Seconds)		Hyperparameter Optimization Method		
		Default Values	GA	Optuna
Penetration	ANN	18.6	1116	672
	XGBoost	6.6	648	426
	RF	14.4	786	522
	SVM	9.6	948	606
Softening point	ANN	19.8	1152	708
	XGBoost	7.2	678	444
	RF	13.8	768	510
	SVM	9.0	930	594
Ductility	ANN	19.2	1134	690
	XGBoost	6.6	660	432
	RF	12.6	738	486
	SVM	8.4	894	570
Viscosity at 135 °C	ANN	18.0	1098	660
	XGBoost	6.0	636	414
	RF	18.6	1116	672
	SVM	6.6	648	426

**Table 3:** Input variables for the first eight samples. Base asphalt types: ES70 and SK70. SBS types: SBS-796, SBS-791H, SBS-792, and SBS-161B. Dosage is in % by weight of base asphalt. Shear temperature in °C, shear rate in rpm, shear time in min, and particle size in μm.

ID	Base Asphalt	Additive	Dosage	Shear Temperature	Shear Rate	Shear Time	Particle Size
1	SK70	SBS-792	7.5	190	6000	60	3.12
2	SK70	SBS-161B	7.5	170	4000	30	9.91
3	ES70	SBS-791H	7.5	180	6000	60	3.97
4	ES70	SBS-792	6	170	6000	30	2.35
5	SK70	SBS-796	6	180	6000	60	2.99
6	ES70	SBS-161B	7.5	190	2000	30	15.00
7	ES70	SBS-796	4.5	170	6000	30	3.45
8	SK70	SBS-792	3	170	2000	120	1.17

**Table 4:** Output variables and FTIR indices for the first eight samples corresponding to Table 1. $I_{PS}$: polystyrene index. $I_{PB}$: polybutadiene index. $I_{B}$: butadiene index. Penetration in 0.1 mm at 25 °C. Softening point in °C. Ductility in cm at 25 °C. Viscosity in Pa·s at 135 °C.

ID	$I_{PS}$	$I_{PB}$	$I_{B}$	Penetration	Softening Point	Ductility	Viscosity at 135 °C
1	449.02	321.65	466.62	43.84	98.03	15.99	4.00
2	321.86	762.89	956.55	45.08	110.88	26.42	4.07
3	461.66	1438.54	538.41	40.19	127.81	45.29	4.93
4	464.85	332.98	363.76	43.35	83.88	19.61	3.29
5	299.08	1851.12	514.30	51.96	109.76	26.47	2.45
6	194.83	461.79	1672.92	44.76	100.22	30.99	2.64
7	122.77	759.83	1011.18	49.46	71.52	28.93	2.02
8	228.44	163.64	901.53	64.08	61.53	8.68	1.18

**Table 5:** Statistical summary of the continuous features and target variables.

Variable	Unit	Mean	Standard Deviation	Minimum	Maximum
Shear temperature	°C	180.0	8.2	170	190
Shear rate	rpm	4000	1633	2000	6000
Shear time	min	70	38.7	30	120
Particle size	μm	4.12	2.87	0.51	11.45
$I_{PS}$	—	0.82	0.51	0.05	1.73
$I_{PB}$	—	4.21	2.89	0.24	8.82
$I_{B}$	—	15.6	11.2	0.79	41.2
Penetration	0.1 mm	56.8	9.5	27.9	79.3
Softening point	°C	69.4	13.8	32.7	98.7
Ductility	cm	24.2	9.8	2.0	48.5
Viscosity at 135 °C	Pa·s	1.83	0.94	0.40	4.93

**Table 6:** Hyperparameter search spaces for Optuna optimization.

Model	Hyperparameter	Search Space	Scale
SVM	C	[0.1, 1000]	Logarithmic
	gamma	[0.001, 10]	Logarithmic
	epsilon	[0.01, 1.0]	Linear
ANN	Hidden layer size	[32, 64, 128, 256]	Discrete
	Learning rate	[0.0001, 0.1]	Logarithmic
	alpha	[0.0001, 0.1]	Logarithmic
RF	*n* estimators	[50, 500]	Discrete
	max depth	[5, 50]	Discrete
	min samples split	[2, 20]	Discrete
	min samples leaf	[1, 10]	Discrete
XGBoost	learning rate	[0.01, 0.3]	Logarithmic
	*n* estimators	[50, 300]	Discrete
	max depth	[3, 15]	Discrete
	sub sample	[0.5, 1.0]	Linear
	colsample bytree	[0.5, 1.0]	Linear

## Data Availability

The data that support the findings of this study are available from the authors upon reasonable request.
